# AC093797.1 as a Potential Biomarker to Indicate the Prognosis of Hepatocellular Carcinoma and Inhibits Cell Proliferation, Invasion, and Migration by Reprogramming Cell Metabolism and Extracellular Matrix Dynamics

**DOI:** 10.3389/fgene.2021.778742

**Published:** 2021-12-03

**Authors:** Xiaoling Liu, Chenyu Wang, Qing Yang, Yue Yuan, Yunjian Sheng, Decheng Li, Suvash Chandra Ojha, Changfeng Sun, Cunliang Deng

**Affiliations:** ^1^ The Department of Infectious Diseases, The Affiliated Hospital of Southwest Medical University, Luzhou, China; ^2^ The Department of Tuberculosis, The Affiliated Hospital of Southwest Medical University, Luzhou, China; ^3^ Laboratory of Infection and Immunity, The Affiliated Hospital of Southwest Medical University, Luzhou, China; ^4^ The Department of Gastroenterology, The Second People’s Hospital of Neijiang, Neijiang, China

**Keywords:** biomarker, HCC, AC093797.1, biological function, long-non-coding RNA

## Abstract

**Purpose:** The risk signature composed of four lncRNA (AC093797.1, POLR2J4, AL121748.1, and AL162231.4.) can be used to predict the overall survival (OS) of patients with hepatocellular carcinoma (HCC). However, the clinical significance and biological function of AC093797.1 are still unexplored in HCC or other malignant tumors. In this study, we aimed to investigate the biological function of AC093797.1 in HCC and screen the candidate hub genes and pathways related to hepatocarcinogenesis.

**Methods:** RT-qPCR was employed to detect AC093797.1 in HCC tissues and cell lines. The role of AC093797.1 in HCC was evaluated *via* the cell-counting kit-8, transwell, and wound healing assays. The effects of AC093797.1 on tumor growth *in vivo* were clarified by nude mice tumor formation experiments. Then, RNA-sequencing and bioinformatics analysis based on subcutaneous tumor tissue was performed to identify the hub genes and pathways associated with HCC.

**Results:** The expression of AC093797.1 decreased in HCC tissues and cell lines, and patients with low expressed AC093797.1 had poor overall survival (OS). AC093797.1 overexpression impeded HCC cell proliferation, invasion, and migration *in vitro* and suppressed tumor growth *in vivo*. Compared with the control group, 710 differentially expressed genes (243 upregulated genes and 467 downregulated genes) were filtered *via* RNA-sequencing, which mainly enriched in amino acid metabolism, extracellular matrix structure constituents, cell adhesion molecules cams, signaling to Ras, and signaling to ERKs.

**Conclusion:** AC093797.1 may inhibit cell proliferation, invasion, and migration in HCC by reprograming cell metabolism or regulating several pathways, suggesting that AC093797.1 might be a potential therapeutic and prognostic marker for HCC patients.

## Introduction

Hepatocellular carcinoma (HCC) causes a considerable number of deaths around the world every year. This has been a challenge in recent years, and it will affect approximately one million people each year by 2025 (Villanueva, 2019; Llovet et al., 2021; Sung et al., 2021). China is one of the countries with the highest incidence and mortality of liver cancer, especially HCC (Chen et al., 2016; Feng et al., 2019). Although many studies have clarified the risk factors of HCC and improved the diagnosis and treatment methods, the 5-year survival rate is still very poor (Greten et al., 2015). The mechanism of tumorigenesis is not yet fully understood, which is a huge obstacle to the treatment of tumors.

Long non-coding RNA (lncRNA) is comprised of a type of non-coding RNA with a length of more than 200 nucleotides, and it plays an important regulatory role in many cellular processes such as epigenetics, cell cycle, and cell differentiation. With deepening research, increasing evidence has confirmed that lncRNAs play a vital role in the biological process of various cancers, including HCC (Zhang et al., 2015; Yang et al., 2020).

In the previous study, we found that LncRNA AC093797.1 (Ensemble ID: ENSG00000233110, also known as: RP11-301L8.2) combined with three lncRNA (POLR2J4, AL121748.1, and AL162231.4) can be used to predict the prognosis of HCC patients by mining the GEO database and analyzing the gene microarray of HCC tissues (Ma and Deng, 2019). However, the biological role of AC093797.1 in liver cancer or any other malignant tumor has not been documented. In this study, we aimed to investigate the biological function of AC093797.1 and predict the key genes or signal pathways on which it is dependent. Finally, we found that the expression of AC093797.1 was decreased in HCC tissues and HCC cells, and patients with higher expression of AC093797.1 tend to have better 5-year survival. Combined with age, BMI, TNM stage, grade, and family-related history, AC093797.1 has good predictive power for the 5-year survival of patients with HCC. In addition, overexpression of AC093797.1 can significantly inhibit the proliferation, invasion, and migration of HCC cells *in vitro* and inhibit tumor growth *in vivo*. AC093797.1 can be involved in the disease process of HCC through a variety of pathways, and that might be a potential diagnostic and therapeutic target.

## Material and Methods

### Specimens and Data Collection

The tumor tissues and paired paracancerous tissues used in this study were obtained from 16 HCC patients who underwent hepatectomy in the department of Hepatobiliary Surgery of the Affiliated Hospital of Southwest Medical University. The specimens were frozen in liquid nitrogen immediately after removal. The histopathological diagnosis of HCC specimens was diagnosed by two pathologists independently, and both were diagnosed as HCC. This study was approved by the Institutional Review Board of the Affiliated Hospital of Southwest Medical University (Luzhou, China). Further, the RNA-seq data and clinical data with survival information were downloaded from UCSC Xena (https://xenabrowser.net/datapages/) to evaluate the predictive power of AC093797.1 and analyze the correlation with clinical indicators.

### Cell Culture and Transfection

HCC cell lines, including HCCLM3, MHCC97-H, Huh7, and HepG2, and human normal liver cells LO2 were purchased from Procell Life Science and Technology Co., Ltd. (Wuhan, China). The overexpression vector of AC093797.1 (OE-AC093797.1) and empty vector (pcDNA3.1) were purchased from Shanghai Generay Biotech Co, Ltd. (Shanghai, China). HCCLM3, MHCC97-H, and Huh7 cells were cultured in DMEM medium (Gibco), LO2 cells were cultured in RPMI 1640 (Gibco), and HepG2 cells were cultured in MEM medium (Gibco). Above media were supplemented with 10% fetal bovine serum (FBS). All the cells were incubated in an incubator with 5% CO_2_ at 37°C.

For the transfection, HCC cells were incubated in a 24-well polypropylene plate and transfected with the empty vector pcDNA3.1 or OE-AC093797.1 according to the instructions of Lipofectamine™ 2000 (Thermo, USA) when the cell confluence reaches about 70–90%. To obtain the stable transfected cell lines, the transfected HCC cells were screened by G418 (1 mg/ml) for 2 weeks.

### RNA Isolation and qPCR

Total RNA of liver tissues or cells was isolated using Trizol reagent. RT first Strand cDNA Synthesis Kit was used to reverse transcribe 2 μg of total RNA into cDNA, and SYBR Green qPCR Master Mix (High ROX) was used for qRT-PCR. All the above reagents were purchased from Servicebio Biotechnology Co., Ltd. (Wuhan, China). The housekeeping gene glyceraldehyde 3-phosphate dehydrogenase (GAPDH) was used as the reference gene, and the relative expression of AC093797.1 was calculated *via* the 2^-△△Ct^ method. The primers used in this study were as follows: GAPDH forward primer: 5′-GGA​CCT​GAC​CTG​CCG​TCT​AG-3′; GAPDH reverse primer: 5′-GTA​GCC​CAG​GAT​GCC​CTT​GA-3′; AC093797.1 forward primer: 5′-TGC​CGC​AAG​GAG​GAG​GCT​ATT​GTT-3′; AC093797.1 reverse primer: 5′-TGG​GAA​GGC​TTA​TTC​ATG​GAC​CTA-3′.

### CCK-8 Assay

Cell-counting Kit-8 (CCK8) reagent was used to determine cell proliferation. Briefly, post-transfection for 24 h, the HCCLM3 cells were seeded into 96-well plates at 3 × 10^3^ cells/well. At 0, 24, 48, 72, and 96 h, 10 μl CCK8 reagent was added to the wells and incubated at 37°C for an extra 4 h, then to detect the absorbance value at 450 nm by a microplate reader.

### Wound-Healing Assay

After 24 h of transfection, the transfected cells were plated into a 24-well plate (2.5 × 10^5^ cells/well). A sterile 200 μl plastic pipette tip was used to make scratches, the image of scratches at 0 and 48 h was captured by an inverted microscope at 40× magnification, and Image J was used to calculate the healing area of scratches.

### Transwell Assays

The 24-well transwell chambers with 8 μm pore size were employed to assess the motility potential of HCC cells. For the invasion experiment, the bottom membrane of the chamber was pre-coated with 50 μl of Matrigel (3 mg/ml). After 24 h of transfection, the transfected cells were harvested and suspended in the serum-free medium at a density of 4 × 10^5^ cells/ml, then 100 μl of cell suspension were dispensed into the upper chamber. Complete medium (600 μl) was added to the lower chamber. After 24 h, adherent cells on the membrane were fixed with paraformaldehyde fixative and stained with 0.1% crystal violet. A cotton swab was used to remove non-migrated cells, the images of 10 random fields at 200× magnification were captured by a microscope, and the number of cells was counted.

### Nude Mice Tumor Formation Experiment

The 5-week-old female BALB/c nude mice weighing 15–20 g were maintained under specific-pathogen-free conditions and randomly divided into control group and OE-AC093797.1 group (*n* = 5 per group). In the nude mice tumor formation experiment, stably transfected HCCLM3 cells (5 × 10^7^ cells in 100 μl) were subcutaneously injected into each nude mice. The mice with tumors were observed, and the volume of the tumor was calculated by the following formula: V_tumor_ = length × width^2^ × π/6. When the tumor grows to about 2000 mm^3^, the mice were sacrificed after anesthesia and tumor tissues were isolated. All procedures for the nude mice experiment were approved by the Animal Care Committee of Southwest Medical University (Luzhou, China).

### Immunohistochemical Assay

Part of the tumor tissue isolated from the nude mice was fixed with 4% paraformaldehyde and used to detect the expression of Ki67 in the tumor tissue according to the instructions of the Ki-67 detection kit (Immunohistochemistry, Sangon Biotech Co., Ltd., Shanghai, China).

### RNA Transcriptome Sequencing and Bioinformatics Analysis

The total RNA was extracted from subcutaneous tumor tissue of nude mice in the OE-AC093797.1 and control group and used to prepare a common transcriptome library; then, the RNA sequencing was performed on the Illumina NovaSeq 6000 platform. The “DNSeq2” package in R 4.0 was used to filter the differentially expressed genes between two groups. |log2foldchange| > 1.5 and adjust *p*-value (FDR) < 0.05 were set as the criterion. Then, gene ontology (GO), KEGG signaling pathway enrichment analysis, and gene set enrichment analysis (GSEA, including c2.cp.kegg v7.4.symbols.gmt and c2.cp.reactome.v7.4.symbols.gmt) were performed. Adjusted *p*-value (FDR) < 0.05 indicates that the gene or the signaling pathway was significantly enriched in the corresponding category. Furthermore, the protein-protein interaction (PPI) network was constructed by the online tools STRING (https://string-db.org/) and visualized by Cytoscape 3.8. Then, the function modules and hub genes were identified by the plunge-in Molecular Complex Detection (MCODE) and cytoHubba using the default parameters and MCC algorithm, respectively.

### Statistical Analysis

All data were expressed as mean ± standard deviation (SD), and the standard two-tailed t-test or one-way analysis of variance (ANOVA) was used to compare the difference between two groups. The correlation between the expression of AC093797.1 and clinical variables was assessed by chi-square test. Univariate and multivariate Cox regression models were used to screen the independent prognostic factor of HCC patients in the software SPSS 26. The subgroup analysis between the expression of AC093797.1 and clinical variables was performed *via* the package “forestplot” in R, and the survival analysis between the expression of AC093797.1 and survival time was performed *via* the package “survival” and “survminer” in R. *P* < 0.05 indicates the differences with statistical significance.

## Results

### LncRNA AC093797.1 was Decreased in HCC and Associated With the Poor Survival of Patients

**FIGURE 1:**
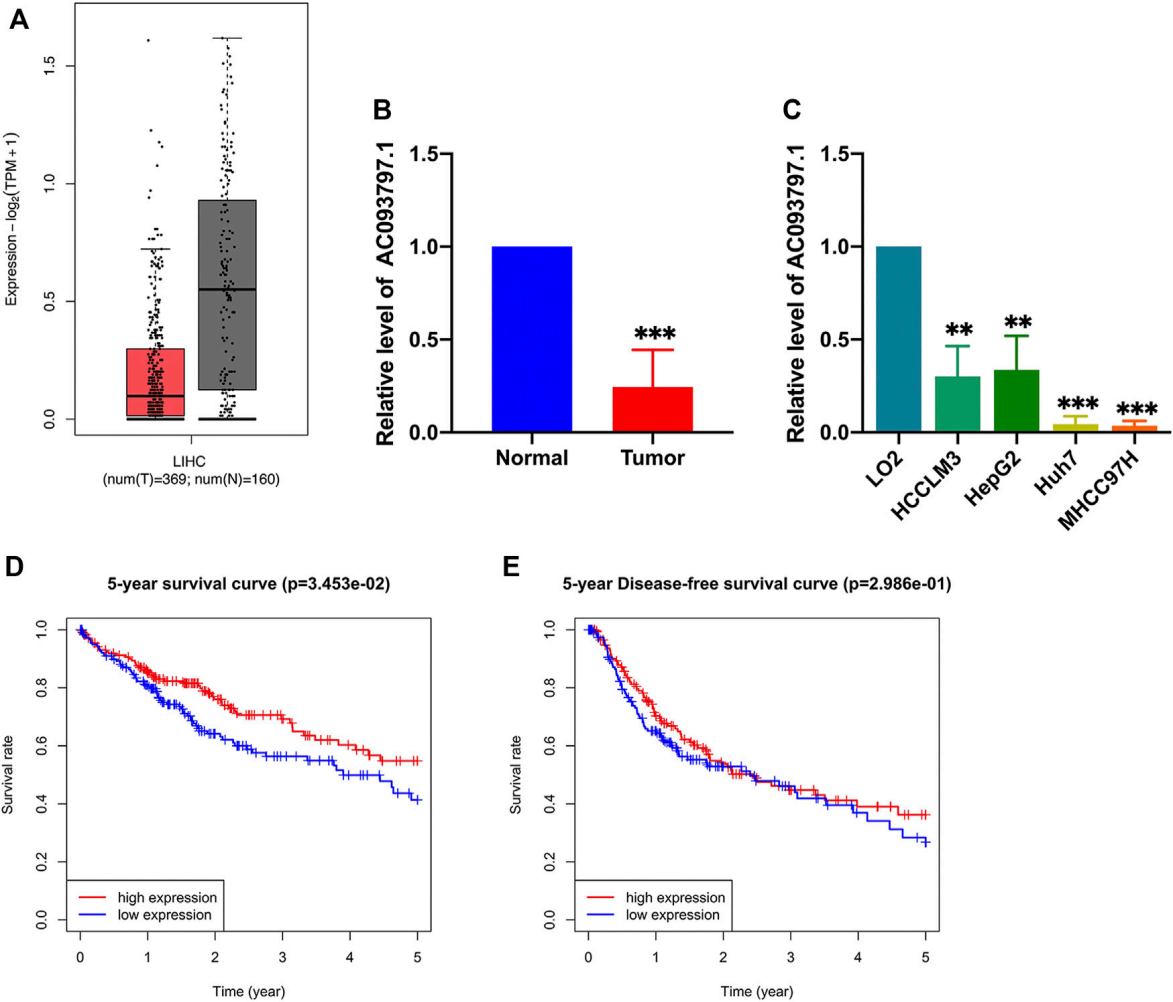
The expression and prognosis value of AC093797.1. **(A)** The expression AC093797.1 in HCC tissues in GEPIA database (red: HCC tissues; grey: normal tissues). **(B)** The expression of AC093797.1 in the 16 HCC tissues collected in this study. **(C)** The expression of AC093797.1 in the HCC cell line HCCLM3, HepG2, Huh7, and MHCC97H. **(D**–**E)** The 5-year survival and DFS plot based on the expression of AC093797.1. **p < 0.01, ***p < 0.001.

The GEPIA database integrates the data from the TCGA and the GTEx database, which recomputed the RNA sequencing raw data based on a standard processing pipeline to minimize differences from distinct sources to make data from different sources more compatible. The GEPIA database contains RNA expression profiles of 369 HCC tissues and 160 normal tissues. Based on the expression profiles of GEPIA, AC093797.1 was significantly low-expressed in HCC tissues than normal liver tissues (*p* < 0.05, Figure 1A). In this study, the expression of AC093797.1 was also confirmed in paired pathological tissues of HCC patients and liver cancer cell lines. As shown in Figures 1B,C, the expression of AC093797.1 in the 16 HCC tissues and four liver cancer cell lines (including HCCLM3, Huh7, HepG2, and MHCC97-H) was significantly decreased than adjacent non-tumor specimens and normal liver cells LO2. Further, the HCC patients with lower expression of AC093797.1 have poorer 5-year survival, but the expression level of the lncRNA was not related to the 5-year disease-free survival (DFS) (Figures 1D,E), indicating that the expression of AC093797.1 may be a biomarker of 5-year survival of HCC patients.

### The Relationship Between AC093709.1 and Clinical Indicators

To explore the correlation between the expression of AC093709.1 and clinical indicators, 365 HCC patients with survival information in the TCGA database were divided into low- and high-expression groups according to the median of AC093797.1. Combined with the 5-year overall survival information, the BMI (>23.9 vs. ≤23.9) and living status of HCC patients were significantly different between the two groups (Table 1), suggesting that the expression level of AC093797.1 may be related to the BMI and 5-year OS of HCC patients. While combined with the 5-year DFS information, the BMI (>23.9 vs. ≤23.9) and hepatic inflammation of HCC patients was significantly different between the two groups (Table 1).

**TABLE 1:** The clinical characters of HCC patients in the AC093797.1 low-/high-expression group.

Clinical features		OS			DFS		
		AC093797.1 expression		*p* value	AC093797.1 expression		*p* value
		High	Low		High	Low	
Age	≤60	81	92	0.161	69	83	0.230
	>60	104	88		84	77	
Gender	Female	66	53	0.119	97	111	0.263
	Male	115	113		56	49	
BMI	≤23.9	66	86	0.045	58	79	0.038
	>23.9	98	82		86	72	
TNM stage	I-II	128	126	0.279	111	116	0.632
	III-IV	38	49		31	37	
Grade	G1/G2	115	115	0.575	95	97	0.570
	G3/G4	61	69		54	63	
Tumor status	Tumor free	80	81	0.550	71	73	0.570
	With tumor	67	55		53	47	
Family history	None	94	110	0.115	80	99	0.080
	Yes	62	45		53	42	
Vascular invasion	None	106	99	0.360	88	87	0.533
	Yes	49	57		44	51	
AFP	≤300 ng/ml	110	102	0.357	112	107	0.291
	>300 ng/ml	29	35		27	35	
Hepatic inflammation	None	70	47	0.067	65	44	0.049
	Yes	55	60		56	48	
Living status	Alive	125	109	0.042	108	100	0.109
	Dead	55	75		44	60	

**FIGURE 2:**
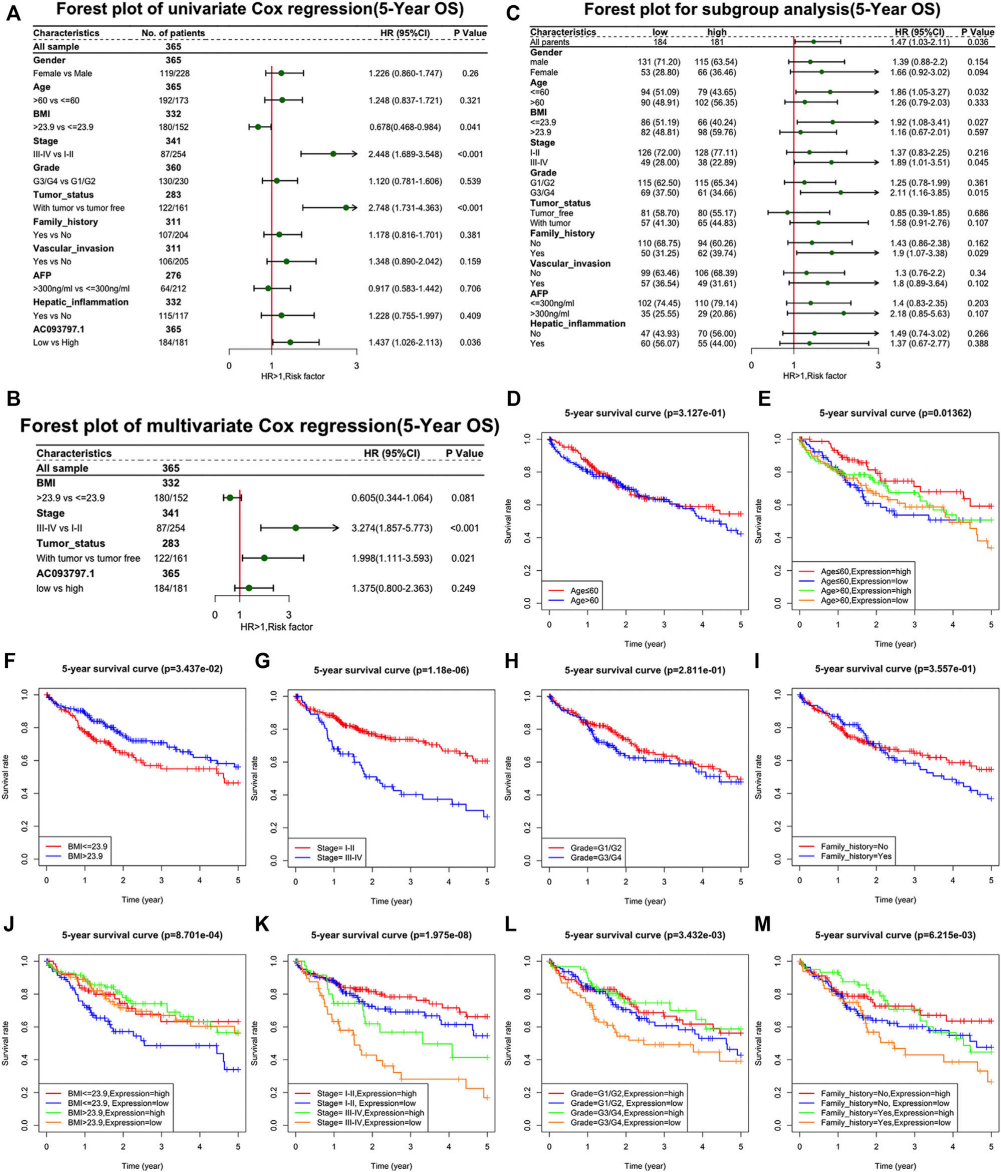
The relationship between AC093709.1 and clinical indicators. **(A)** Forest plot of univariate Cox regression (5-year overall survival, OS). **(B)** Forest plot of multivariate Cox regression (5-year OS). **(C)** Forest plot for subgroup analysis (5-year OS). **(D–M)** The survival plot of HCC patients is based on age, BMI, TNM stage, grade, family-related history, and the expression of AC093797.1.

Further, univariate Cox regression and multivariate Cox regression analyses were performed to identify the risk factors that affect the prognosis of HCC patients. As shown in Figure 2A, the BMI ≤23.9, TNM stage Ⅲ-Ⅳ, with tumor, and the low expression of AC093797.1 were the risk factors of 5-year survival. The low expression of AC093797.1 was not acting as a risk factor of 5-year DFS, and the risk factors of 5-year DFS of HCC patients were the TNM stage Ⅲ-Ⅳ, with tumor and vascular invasion (Supplementary Figures S1A, B). However, the expression level of AC093797.1 was not an independent risk factor that affects the 5-year OS (Figure 2B), implying that the expression of AC093797.1 may interact with other clinical indicators to affect the 5-year survival of HCC patients.

The results of subgroup analysis showed that the expression of AC093797.1 was an independent risk factor affecting 5-year survival in patients with age ≤60, BMI ≤23.9, related family history, TNM stage III-IV, or grade III-IV (Figure 2C). But the expression of AC093797.1 was not an independent risk factor affecting 5-year DFS in any subgroup (Supplementary Figure S2C).

Besides, the expression of AC093797.1 combined with age, BMI, TNM stage, grade, and related family history can better predict the 5-year survival in patients with age ≤ 60, BMI ≤ 23.9, related family history, TNM stage III-IV, or grade III-IV (Figures 2D–M).

It was speculated that the expression of AC093797.1 may have a significant clinical correlation with clinical indicators in specific HCC patients.

### AC093797.1 Inhibited Cell Proliferation, Migration, and Invasion of the HCC Cells

**FIGURE 3:**
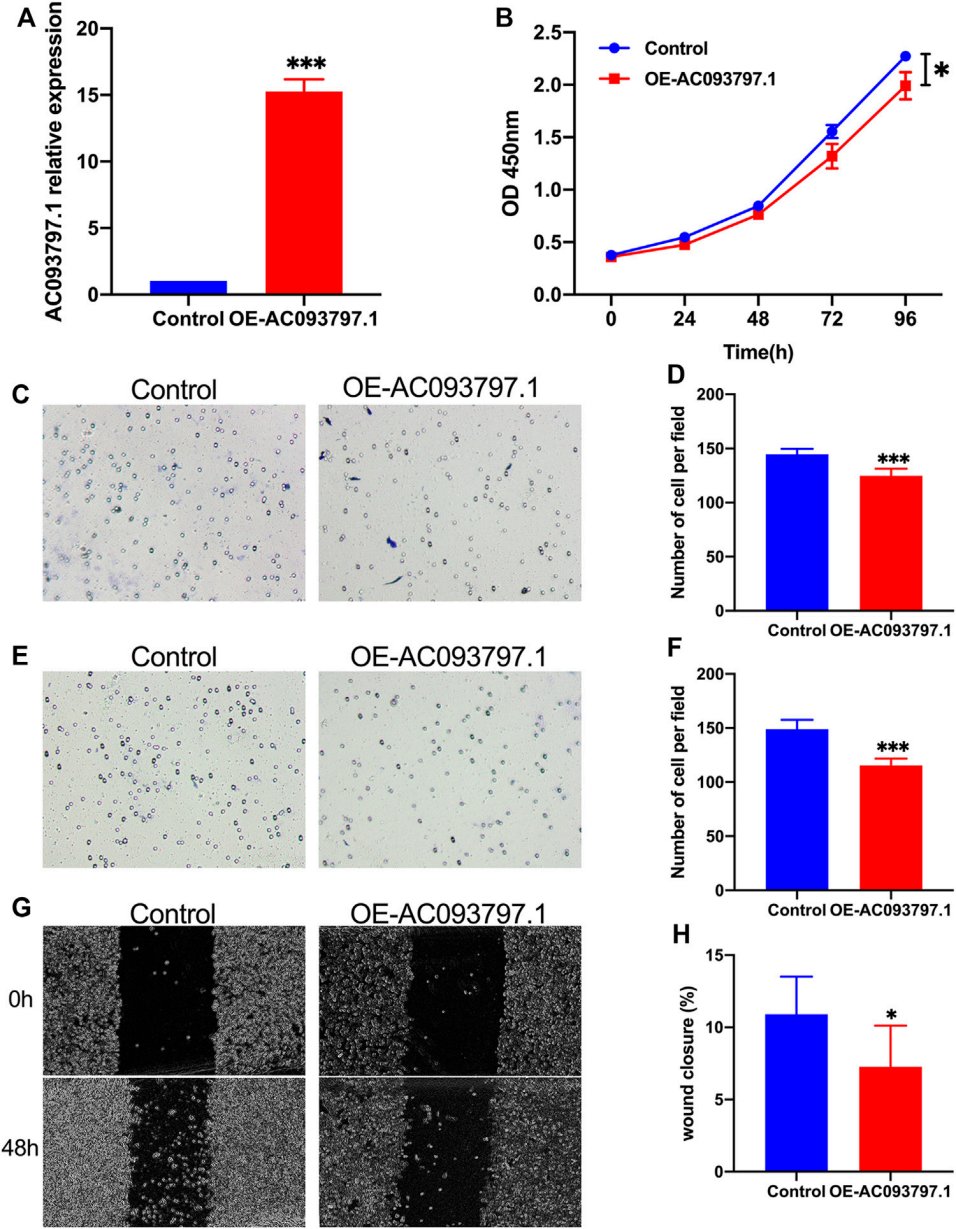
AC093797.1 inhibited cell proliferation, migration, and invasion of the HCC cells. **(A)** The expression of AC093797.1 in the HCC cells HCCLM3 transfected with OE-AC093797.1 or empty vector. **(B)** CCK8 assay detect the proliferation of HCCLM3 transfected with OE-AC093797.1 or empty vector. **(C, D)** The invasion assay of HCCLM3 transfected with OE-AC093797.1 or empty vector. **(E, F)** The migration assay of HCCLM3 transfected with OE-AC093797.1 or empty vector. **(G**–**H)** The wound-healing assay of HCCLM3 transfected with OE-AC093797.1 or empty vector. *p < 0.05, ***p < 0.001.

To further clarify the biological role of AC093797.1 in HCC, the overexpression vector OE-AC093797.1 was transfected into HCCLM3 cells. The expression of AC093797.1 in the transfected HCCLM3 was detected by qPCR. As shown in Figure 3A, the expression of AC093797.1 was significantly increased in the cells transfected with OE-AC093797.1. Through CCK8, transwell, and wound healing assays, it was found that the overexpressed AC093797.1 in the HCCLM3 cells could inhibit cell proliferation (Figure 3B), migration (Figures 3C,D, G–H), and invasion (Figures 3E,F).

### AC093797.1 Overexpression Inhibits the Tumor Growth *in vivo*

**FIGURE 4:**
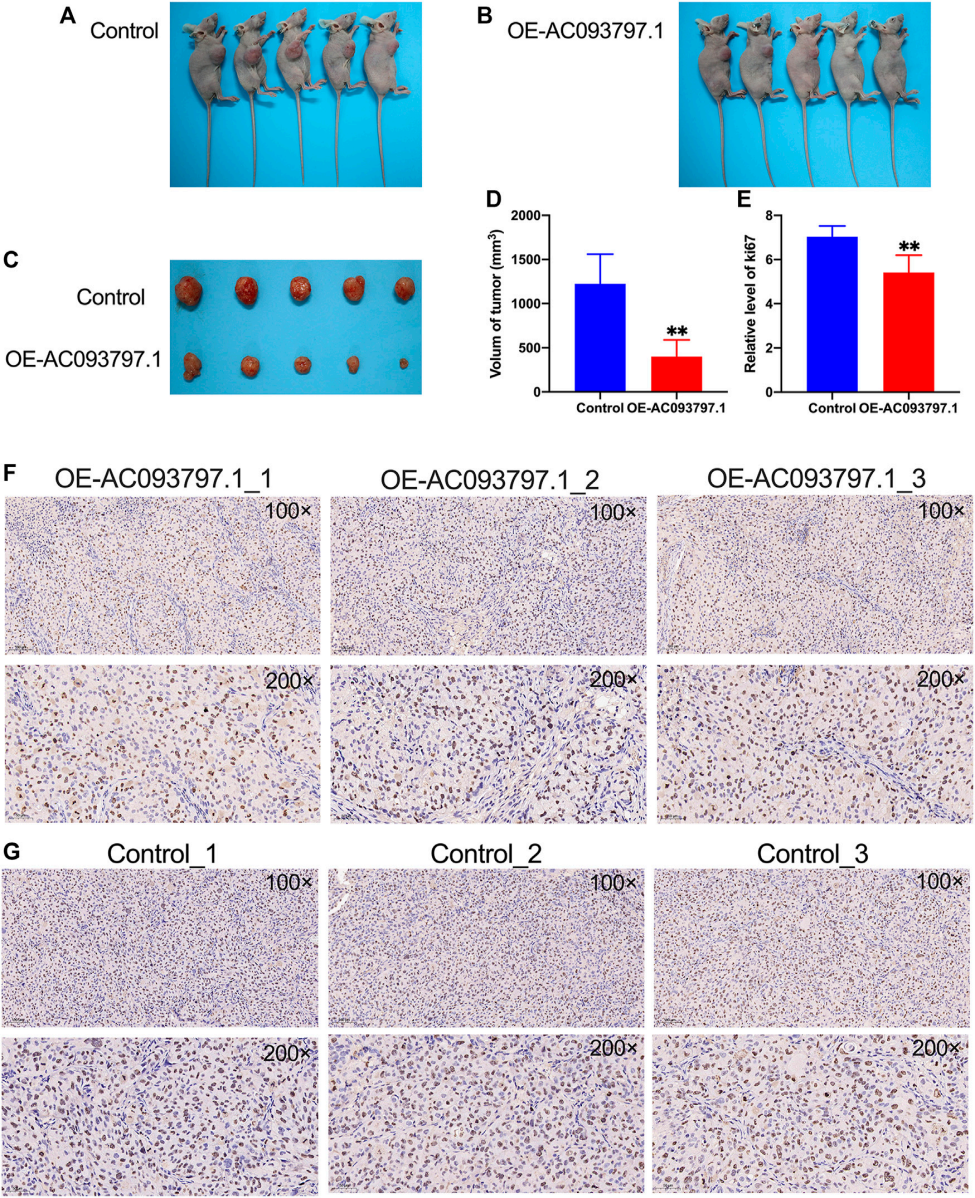
AC093797.1 can inhibit the growth of tumors in nude mice. **(A, B)** The nude mice with tumors in the OE-AC093797.1 group and control group. **(C)** The tumors isolated from the mice in the OE-AC093797.1 group and control group. **(D)** The volume of tumors isolated from the mice in the OE-AC093797.1 group and control group. **(E)** The percentage of positive straining area of Ki67 in the OE-AC093797.1 and control group. **(F, G)** The expression of Ki67 in the tumors isolated from OE-AC093797.1 and control group detected by immunohistochemical assay. **p < 0.01.

*Via* the nude mice tumor formation experiment, it was found that the volume of the tumor isolated from the OE-AC093797.1 group was significantly smaller than the control group on the 45th day after subcutaneous injection (Figures 4A–D), which suggested that high expression of AC093797.1 can inhibit the growth of tumors formed by HCCLM3 *in vivo*. Ki67 protein is a nuclear protein strictly associated with cell proliferation, and it is generally believed that the higher the expression level of Ki67, the faster the tumor growth, the lower the degree of differentiation, and the worse the prognosis of tumor patients. The immunohistochemical results of ki67 showed that the positive staining area in the tumor tissue of the OE-AC097997.1 group was significantly lower compared with the control group (Figures 4E–G), indicating that AC093797.1 significantly weakened the cell division of HCC cells *in vivo*.

### Bioinformatics Analysis Based on Subcutaneous Tumor Specimens

**FIGURE 5:**
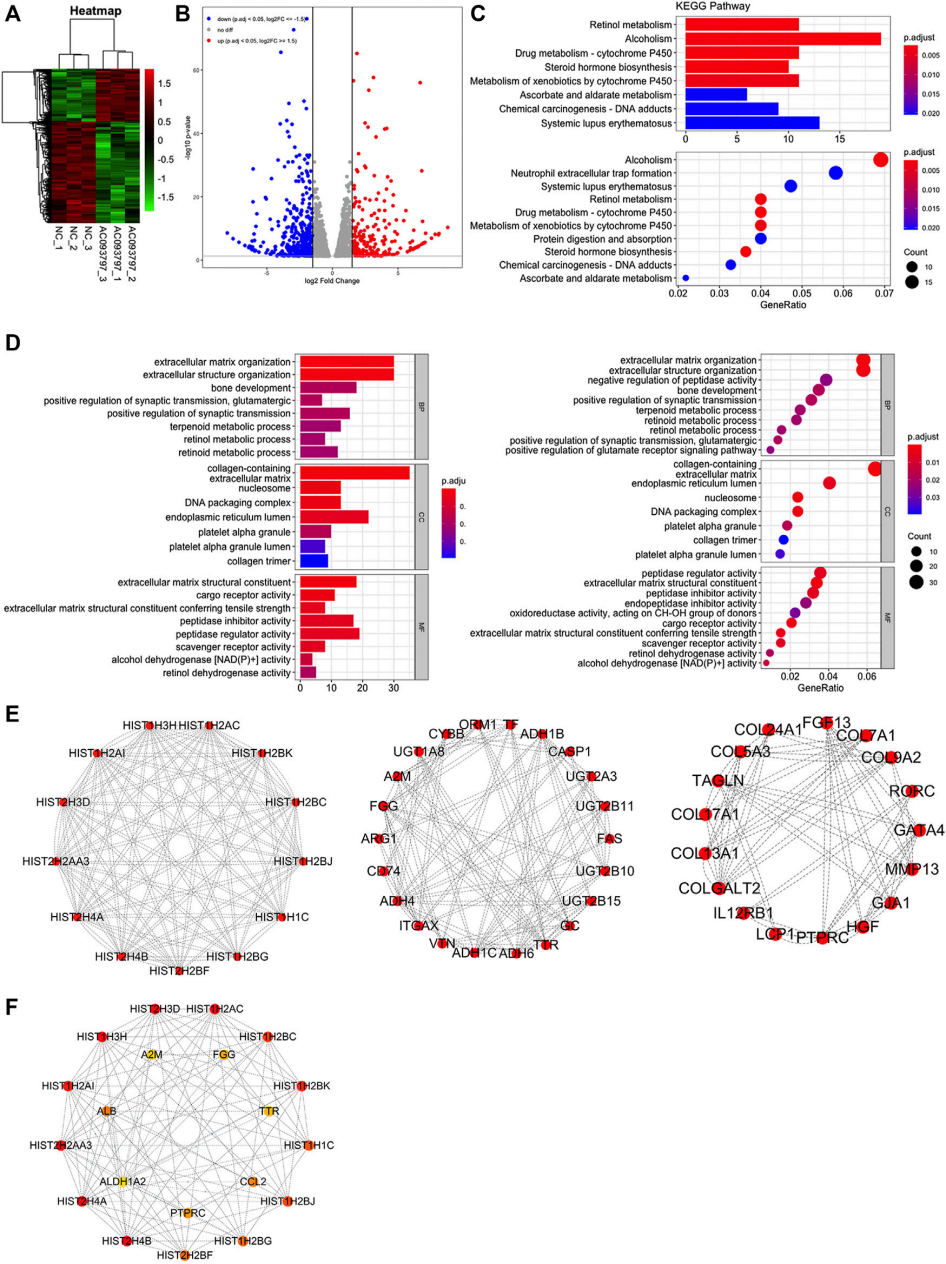
Bioinformatics analysis based on subcutaneous tumor specimens. **(A, B)** The heatmap and volcano plot of the differentially expressed genes between OE-AC093797.1 group and control group. **(C)** The bar and dot figures of KEGG of the differentially expressed genes. **(D)** The bar and dot figures of GO of the differentially expressed genes. **(E)** Three function module of protein-protein interaction (PPI) network. **(F)** The top20 core genes of PPI network.

In this study, subcutaneous tumor tissues of three nude mice in the OE-AC093797.1 and control group were used to extract the total RNA and performed RNA sequencing independently. Then, the differentially expressed genes of the OE-AC097997.1 group and control group were filtered by the package “DESeq2” in R. The result showed that 710 differentially expressed genes were identified, including 243 upregulated genes and 467 downregulated genes (Figures 5A,B, Supplementary Table S1).

**FIGURE 6:**
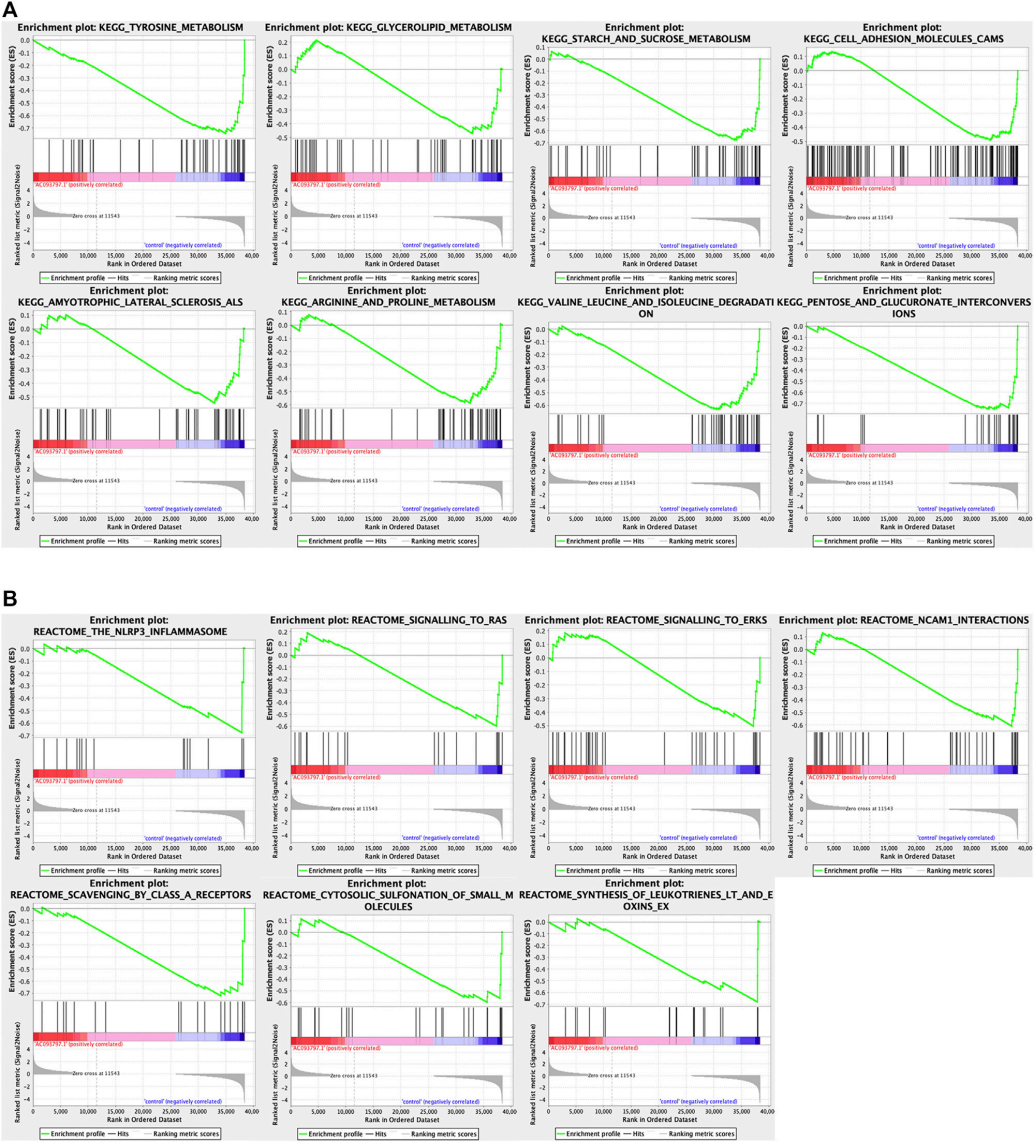
The GSEA analysis between the OE-AC093797.1 and control group. **(A)** GSEA analysis based on the “c2.cp.kegg v7.4.symbols.gmt”. **(B)** GSEA analysis based on the “c2.cp.reactome.v7.4.symbols.gmt”.

To explore the involved biological processes and functions of these differentially expressed genes, the GO, KEGG signal pathway enrichment analyses, and GSEA were conducted. The GO analysis contained three terms, including biological process (BP), cellular component (CC), and Molecular Function (MF). For the BP, the differentially expressed genes were significantly enriched in the extracellular matrix organization, extracellular structure organization, etc.; for the CC, the differentially expressed genes were significantly enriched in the collagen-containing extracellular matrix, endoplasmic reticulum lumen, nucleosome, etc.; for the MF, the differentially expressed genes were significantly enriched in peptidase regulator activity, extracellular matrix structure constituent, etc. (Figure 5C). *Via* the KEGG enrichment analysis, the differentially expressed genes were significantly enriched in retinal metabolism, alcoholism, drug metabolism-cytochrome P450, steroid hormone biosynthesis, metabolism of xenobiotics by cytochrome P450, etc. (Figure 5B). The result of GSEA showed that the KEGG pathway gene set of the metabolism of glycolipid metabolism, tyrosine metabolism, starch and sucrose metabolism, arginine and proline metabolism, valine leucine and isoleucine degradation, cell adhesion molecules cams, pentose and glucuronate interaction, amyotrophic lateral sclerosis (Figure 6A), and the Reactome pathway gene set of the NLRP3 inflammasome, signaling to Ras, and signaling to ERKs, and other three pathway was significantly enriched in the control group (Figure 6B).

Furthermore, a PPI network was constructed in the STRING database and visualized by the Cytoscape. By the MCODE plug-in in Cytoscape, three function modules of the PPI network were parsed out (Figures 5E–G), and the top 20 core genes were extracted from the PPI network *via* the plug-in CytoHubba using the MCC algorithms (Figure 5H). Combining the above two algorithms, 13 histone family members HIST1H2BJ, HIST1H2BK, HIST1H2AC, HIST2H2AA3, HIST1H2AI, HIST1H2BG, HIST2H4B, HIST2H4A, HIST1H2BC, HIST1H1C, HIST1H3H, HIST2H3D, HIST2H2BF, and TTR, A2M, PTPRC, and FGG were selected as hub genes finally.

## Discussion

According to the newest report from World Health Organization (WHO), among all malignant tumors, the incidence rate of liver cancer patients ranks sixth and the mortality rate ranks third (Valery et al., 2018; Sung et al., 2021). The stages of the tumor determine the prognosis of liver cancer generally, and the 5-year survival of patients with early-stage liver cancer can be more than 70%. In contrast, patients with advanced symptoms who need systemic therapy only had a median survival of 1–1.5 years (Villanueva, 2019). At present, the main treatment method for liver cancer is surgery, and this treatment also makes the early-stage patients obtain the maximum benefit. Consistent with clinical observation, we found that the TNM stage was one of the independent risk factors that affect the 5-year OS and DFS of HCC patients, and patients at stage Ⅲ-Ⅳ have a worse prognosis.

Nonetheless, other clinical indicators, including age, gender, grade, related family history, etc., are also factors that cannot be ignored in clinical practice that affect the prognosis of HCC patients. However, those indicators were not risk factors to the 5-year OS and DFS based on the survival information of HCC patients in the TCGA database. The lncRNA AC093797.1 was downregulated in HCC, and the HCC patients with lower expression of the lncRNA have a poorer prognosis. *Via* subgroup analysis, we found that the expression of AC093797.1 was an independent risk factor affecting 5-year survival in patients with age ≤ 60, BMI ≤ 23.9, related family history, TNM stage III-IV, or grade III-IV, and AC093797.1 combined with age, BMI, TNM stage, grade, and related family history can better predict the 5-year survival in these specific patients, indicating that AC093797.1 may have a close correlation with these clinical indicators and more suitable as a prognostic marker in those specific HCC patients.

Until now, the biological function of AC093797.1 has not been reported in liver cancer or any other malignant tumors. To explore the role of AC093797.1 in the HCC, we transformed the overexpression vector OE-AC093797.1 into HCCLM3, a liver cancer cell line with high spontaneous lung metastasis. In the CCK8, transwell, and wound healing assays, overexpressed AC093997.1 showed inhibitory effects on cell proliferation, invasion, and migration. This inhibitive tumorigenicity role of AC093797.1 was also confirmed by the nude mice tumor formation experiment, the result showed that the volume of tumors in the OE-AC093797.1 group was smaller than the control group, and the decreased Ki67 also revealed the weakened cell proliferation of tumors in the OE-AC093797.1 group. However, no lung metastases were seen in all the mice examined.

Considering that the research on AC093797.1 is very limited, we performed RNA sequencing on tumor tissues isolated from nude mice to gain a broader understanding of the regulatory role of AC093797.1 in HCC. Finally, we obtained 710 differentially expressed genes between the cells transfected with OE-AC093797.1 or empty vector, including 243 upregulated and 467 downregulated genes. *Via* the GO, KEGG, and GSEA enrichment analysis, we found that those differential genes were mainly significant enriched in the following KEGG signaling pathway: the retinal metabolism, alcoholism, drug metabolism-cytochrome P450, steroid hormone biosynthesis, metabolism of xenobiotics by cytochrome P450, etc., and mainly involved in the extracellular matrix organization, extracellular structure organization, and other biology process or molecular function. Extracellular matrix (EMC) is the major component of the local microenvironment or niche of cancer. The importance of ECM to cancer progression is now well recognized (Poltavets et al., 2018). The abnormal ECM dynamics are a hallmark of cancer (Lu et al., 2012) and lead to the development of cancer (Walker et al., 2018). The upregulated matrix metallopeptidase, including MMP-1, −3, −7, −10, −11, −13, −14, −16, −26, and −28, are favoring the invasion and metastasis (Scheau et al., 2019), while MMP1, MMP13, and MMP28 were significantly downregulated in the AC093797.1 overexpressed HCC cell HCCLM3, suggesting that this may be one of the reasons AC093797.1 inhibited the migration and invasion of HCC cells.

It was worth noting that the GSEA enrichment analysis using the expression matrix found that KEGG pathway gene set of the metabolism of glycolipid, tyrosine, starch and sucrose, arginine and proline, valine leucine and isoleucine degradation, cell adhesion molecules cams, pentose and glucuronate interaction, and amyotrophic lateral sclerosis were significantly enriched in the control group.

The rapid growth of cancer cells usually runs out of glucose and must use other raw materials as substitution, such as fats and amino acids (Nguyen et al., 2020). Tyrosine is one of the preferred raw materials for cancer, but it is rarely used by normal healthy cells (Nguyen et al., 2020). The catabolism of all three essential amino acids valine, isoleucine, and leucine can also yield NADH and FADH2 which can be utilized for ATP generation. The process of arginine metabolism can generate two derivatives (glutamine and proline) with different functions in cancer development (Zou et al., 2019). The rapid tumor growth has a high demand for glutamine, although it is a non-essential amino acid (Zou et al., 2019). Cancer cells prefer to rely on aerobic glycolysis rather than the oxidation of pyruvate to meet the rapid proliferation of the high energy demand (Zhou et al., 2021). Zhou et al. found that the oroxyloside (OAG)-induced glycolipid metabolic switch could increase ROS levels and lead to G1 cell cycle arrest and growth inhibition of HCC cells (Zhou et al., 2021). Pentose and glucuronate interconversions were deregulated in 100% cancer (Becker et al., 2012). The deregulated pentose and glucuronate interconversions were not enriched in the HCC cells transfected with OE-AC093797.1. It seems that AC073797.1 might alleviate the effect of this pathway on HCC cells. The above results reveal that cells transfected with OE-AC093797.1 seem to have less energy and biomaterial supply than the cells in the control group or reprogramed the metabolic process, and result in weaker proliferation ability of the cells transfected with OE-AC093797.1.

In addition, the Reactome pathway gene set of the NLRP3 inflammasome, signaling to Ras, and signaling to ERKs and other three pathways was shown. It has been reported that NLRP3 inflammasome has different effects in different malignancies and is considered a double-edged sword against cancer (Ju et al., 2021). But the role of NLRP3 inflammasome activation in HCC remains unclear. The Ras/MAPK pathway is activated in 50–100% of human HCC and is associated with poor prognosis (Delire and Stärkel, 2015). Ras is the first intracellular effector of the ERK1/2 pathway. Various extracellular stimuli can trigger the transformation of RAS from an inactive form to an active form and then through multiple effectors to activate the pathways implicated in cell growth, survival, differentiation, and migration (Delire and Stärkel, 2015). Ras/MAPK is usually activated in more than half of HCC patients and is associated with a poor prognosis (Delire and Stärkel, 2015). Ras/MAPK pathway effectors are considered potential targets for the treatment of HCC.

Besides, we also analyzed the interaction between the differentially expressed genes *via* Cytoscape and identified 13 histones and TTR, A2M, PTPRC, and FGG as the hub genes. Although these histones and other hub genes are mostly used as prognostic markers of tumor patients, a few studies have shown that histones may be related to the malignancy of tumor cells. In low-grade glioma (LGG), the upregulated HIST1H2BK was an indicator of poor prognosis and may be a promising biomarker for the treatment of LGG (Liu et al., 2020). HIST1H2AI might involve in nucleosome assembly and DNA packaging (Han et al., 2014). Upregulated HIST1HABF can enhance the cancer stem cell phenotype, malignancy, and liver metastasis through the activation of Notch signaling in colorectal carcinoma (Qiu et al., 2021). In adrenocortical carcinoma, the hub gene HIST1H1C was associated with poor overall survival (Alshabi et al., 2019). For other hub genes, transthyretin (TTR) has the ability to stimulate tumor growth through regulation of tumor, immune, and endothelial cells (Lee et al., 2019); FGG is capable of promoting migration and invasion in hepatocellular carcinoma cells through activating epithelial to mesenchymal transition (EMT) (Zhang et al., 2019); and PTPRC can interact with CXCR4 in a putative molecular network constructed from microarray data, which is closely related to colon cancer metastasis (Chu et al., 2017).

## Conclusion

In this study, we found that AC093797.1 was downregulated in the HCC tissues and four HCC cell lines, and low expression of AC093797.1 in HCC patients was associated with a poor prognosis. AC093797.1 overexpression may inhibit tumor growth in nude mice and inhibits cell proliferation, invasion, and migration *in vitro*. The differentially expressed genes identified by RNA-sequencing are mostly involved in the cell division or metastatic pathways. In summary, our research suggests that AC093797.1 may be a promising diagnostic and therapeutic target for hepatocellular cancer.

## Data Availability

The RNA-sequencing data presented in the study were deposited in the Gene Expression Omnibus (GEO) repository, accession number GSE186933 (https://www.ncbi.nlm.nih.gov/geo/query/acc.cgi?acc=GSE186933).
