# Supplementary material for: AC093797.1 as a Potential Biomarker to Indicate the Prognosis of Hepatocellular Carcinoma and Inhibits Cell Proliferation, Invasion, and Migration by Reprogramming Cell Metabolism and Extracellular Matrix Dynamics
Source: Front Genet. 2021 Dec 3;12:778742. doi: 10.3389/fgene.2021.778742 (PMC8678093; doi:10.3389/fgene.2021.778742)
Supplement: Supplementary file 1 [file Table1.docx]

| **Supplementary table 1. The differential expressed genes between OE-AC093797.1 and control group (OE-AC093797.1 vs control)** | | | | |
| --- | --- | --- | --- | --- |
| gene_id | log2FoldChange | pvalue | padj | gene_name |
| ENSG00000175899 | -6.3257585 | 8.33E-06 | 0.00011552 | A2M |
| ENSG00000183044 | -2.111847 | 1.61E-08 | 4.13E-07 | ABAT |
| ENSG00000154262 | -2.9200987 | 0.00014046 | 0.00135331 | ABCA6 |
| ENSG00000140798 | 4.98156305 | 0.00060272 | 0.00474773 | ABCC12 |
| ENSG00000278922 | -1.9742866 | 0.00024475 | 0.00218594 | AC002310.6 |
| ENSG00000253308 | -2.6595855 | 0.00436073 | 0.02432713 | AC004080.1 |
| ENSG00000258472 | -2.5111452 | 8.61E-37 | 6.53E-34 | AC005726.1 |
| ENSG00000264608 | -1.9854698 | 3.27E-10 | 1.14E-08 | AC005726.3 |
| ENSG00000250770 | -2.634809 | 9.90E-05 | 0.00100203 | AC005865.2 |
| ENSG00000285523 | 1.76905576 | 0.00560403 | 0.02969045 | AC005906.3 |
| ENSG00000268460 | -2.545989 | 0.00290005 | 0.01745102 | AC006262.1 |
| ENSG00000233191 | -3.9101332 | 0.00065565 | 0.00510289 | AC006372.2 |
| ENSG00000261342 | -2.5755797 | 0.00214139 | 0.01368984 | AC006538.1 |
| ENSG00000260573 | -3.0457054 | 0.00405528 | 0.02293668 | AC007493.1 |
| ENSG00000263096 | -5.6008121 | 0.00030287 | 0.00262148 | AC007638.2 |
| ENSG00000229689 | 1.79895244 | 2.57E-06 | 4.04E-05 | AC009237.3 |
| ENSG00000269473 | -2.0670863 | 0.00163052 | 0.01094219 | AC012313.8 |
| ENSG00000248363 | 6.48085948 | 4.82E-06 | 7.13E-05 | AC016550.2 |
| ENSG00000229413 | -2.0674207 | 0.00359209 | 0.02083096 | AC018638.1 |
| ENSG00000236283 | -3.2745655 | 3.21E-06 | 4.94E-05 | AC019197.1 |
| ENSG00000254119 | 3.98412959 | 4.19E-45 | 4.24E-42 | AC025524.2 |
| ENSG00000232353 | 4.37122224 | 2.65E-05 | 0.00031833 | AC026320.1 |
| ENSG00000261474 | -1.7637426 | 0.00171156 | 0.01138542 | AC026471.4 |
| ENSG00000248187 | 1.71357461 | 8.17E-06 | 0.00011361 | AC078850.1 |
| ENSG00000285744 | -2.5206107 | 6.99E-05 | 0.00074407 | AC083837.2 |
| ENSG00000254054 | 2.68328969 | 7.94E-13 | 4.52E-11 | AC087273.2 |
| ENSG00000167912 | 1.99630322 | 0.0001782 | 0.00166506 | AC090152.1 |
| ENSG00000251127 | 1.92434756 | 1.82E-05 | 0.00022609 | AC091173.1 |
| ENSG00000257752 | 3.70652796 | 0.00760825 | 0.03808306 | AC091516.1 |
| ENSG00000260417 | 5.956089 | 9.13E-06 | 0.00012565 | AC092127.1 |
| ENSG00000240859 | 2.19389084 | 0.00904068 | 0.04369602 | AC093627.4 |
| ENSG00000250376 | -1.9917287 | 1.45E-09 | 4.50E-08 | AC093720.1 |
| ENSG00000223652 | 6.24274025 | 1.29E-05 | 0.00016894 | AC106786.1 |
| ENSG00000250038 | -4.113465 | 2.05E-13 | 1.29E-11 | AC109588.1 |
| ENSG00000250696 | -1.5034536 | 0.00105926 | 0.0076586 | AC111000.4 |
| ENSG00000249763 | -2.4842768 | 0.00267848 | 0.01640399 | AC114786.1 |
| ENSG00000250612 | -1.8975939 | 1.02E-08 | 2.72E-07 | AC114786.2 |
| ENSG00000258352 | 1.67452752 | 0.00325211 | 0.01921753 | AC119044.1 |
| ENSG00000279641 | -2.7528065 | 0.00551764 | 0.02933511 | AC120057.3 |
| ENSG00000253414 | -2.5551529 | 0.00895379 | 0.0434173 | AC124067.2 |
| ENSG00000254290 | -2.2833612 | 1.69E-14 | 1.24E-12 | AC124067.4 |
| ENSG00000166104 | -9.1786077 | 4.89E-14 | 3.40E-12 | AC126323.1 |
| ENSG00000259878 | -5.0556462 | 0.00065675 | 0.00510715 | AC126323.3 |
| ENSG00000261296 | -5.4569122 | 2.65E-06 | 4.16E-05 | AC126323.6 |
| ENSG00000253366 | 3.45869902 | 0.00630857 | 0.03270046 | AC139272.1 |
| ENSG00000285722 | 2.61260533 | 2.98E-06 | 4.63E-05 | AC207130.1 |
| ENSG00000271698 | -2.992976 | 0.00786755 | 0.03920863 | AC233992.2 |
| ENSG00000281571 | -1.632228 | 0.00021127 | 0.0019256 | AC241585.2 |
| ENSG00000177076 | -2.9305931 | 3.57E-10 | 1.23E-08 | ACER2 |
| ENSG00000144476 | 1.55583664 | 1.13E-12 | 6.11E-11 | ACKR3 |
| ENSG00000129048 | -1.9175182 | 3.35E-05 | 0.00038959 | ACKR4 |
| ENSG00000102575 | -2.7900048 | 7.00E-12 | 3.35E-10 | ACP5 |
| ENSG00000183549 | 2.13079635 | 0.00382306 | 0.02188382 | ACSM5 |
| ENSG00000111058 | -2.137998 | 2.35E-05 | 0.00028515 | ACSS3 |
| ENSG00000169067 | -3.2533645 | 0.00019157 | 0.00177 | ACTBL2 |
| ENSG00000123612 | 2.28774437 | 0.00051459 | 0.00415134 | ACVR1C |
| ENSG00000249162 | -2.8999797 | 0.00580779 | 0.0305567 | ADAM20P3 |
| ENSG00000166106 | 4.46272474 | 3.01E-28 | 9.99E-26 | ADAMTS15 |
| ENSG00000184060 | -1.726675 | 0.00373881 | 0.02153008 | ADAP2 |
| ENSG00000111452 | -2.4048195 | 0.00014422 | 0.00138354 | ADGRD1 |
| ENSG00000069122 | -3.6798077 | 7.72E-11 | 3.07E-09 | ADGRF5 |
| ENSG00000144820 | -1.9182808 | 0.00954067 | 0.04563692 | ADGRG7 |
| ENSG00000150471 | -2.2129987 | 4.55E-14 | 3.19E-12 | ADGRL3 |
| ENSG00000196616 | -5.8272556 | 3.54E-13 | 2.16E-11 | ADH1B |
| ENSG00000248144 | -3.492286 | 5.43E-22 | 9.70E-20 | ADH1C |
| ENSG00000198099 | -4.6422207 | 5.46E-29 | 1.88E-26 | ADH4 |
| ENSG00000172955 | -1.6195461 | 5.26E-16 | 4.63E-14 | ADH6 |
| ENSG00000224420 | -1.6044637 | 0.00340806 | 0.01995333 | ADM5 |
| ENSG00000128271 | -2.7810367 | 0.00426081 | 0.02390238 | ADORA2A |
| ENSG00000178803 | -1.7229726 | 9.78E-05 | 0.00099192 | ADORA2A-AS1 |
| ENSG00000170214 | 1.57941725 | 2.80E-10 | 9.95E-09 | ADRA1B |
| ENSG00000235609 | 1.58093085 | 0.00020185 | 0.00185367 | AF127577.4 |
| ENSG00000272620 | 3.12880781 | 6.72E-62 | 2.45E-58 | AFAP1-AS1 |
| ENSG00000106351 | -1.9085266 | 4.93E-34 | 2.57E-31 | AGFG2 |
| ENSG00000173467 | -2.1501573 | 0.00020728 | 0.00189851 | AGR3 |
| ENSG00000226812 | -1.5573899 | 0.00239444 | 0.01500749 | AL117382.1 |
| ENSG00000279164 | 3.6542631 | 0.00969342 | 0.0462583 | AL118508.3 |
| ENSG00000258927 | 2.5931913 | 0.00014584 | 0.0013956 | AL133467.2 |
| ENSG00000270038 | 4.03235249 | 0.00024909 | 0.00221387 | AL133467.4 |
| ENSG00000278445 | -2.3975367 | 0.00281292 | 0.01704968 | AL137246.2 |
| ENSG00000230570 | 3.76526618 | 0.00628344 | 0.03259803 | AL139095.3 |
| ENSG00000260912 | -2.8800666 | 5.38E-16 | 4.72E-14 | AL158206.1 |
| ENSG00000275216 | 2.48343006 | 0.00017207 | 0.00161523 | AL161431.1 |
| ENSG00000277496 | -1.8431574 | 2.60E-05 | 0.0003122 | AL357033.4 |
| ENSG00000236366 | -2.0138279 | 0.00276726 | 0.01684736 | AL359313.1 |
| ENSG00000233593 | -1.7608299 | 0.00045613 | 0.00374864 | AL590094.1 |
| ENSG00000163631 | -1.9285483 | 2.30E-11 | 1.01E-09 | ALB |
| ENSG00000128918 | -1.5553763 | 0.00572762 | 0.03021344 | ALDH1A2 |
| ENSG00000136010 | 2.32349335 | 1.02E-09 | 3.26E-08 | ALDH1L2 |
| ENSG00000230002 | 1.97096325 | 0.0081927 | 0.04047477 | ALMS1-IT1 |
| ENSG00000104899 | -1.5778586 | 5.09E-13 | 3.04E-11 | AMH |
| ENSG00000166025 | 3.01198928 | 0.00274748 | 0.01677019 | AMOTL1 |
| ENSG00000240038 | -2.1654004 | 4.27E-09 | 1.22E-07 | AMY2B |
| ENSG00000101280 | 6.11786693 | 3.36E-05 | 0.00038963 | ANGPT4 |
| ENSG00000136859 | -1.7975322 | 1.64E-13 | 1.05E-11 | ANGPTL2 |
| ENSG00000132855 | -1.7124455 | 0.00282621 | 0.01710784 | ANGPTL3 |
| ENSG00000089847 | -1.5565049 | 1.22E-05 | 0.00016279 | ANKRD24 |
| ENSG00000198483 | -1.7798173 | 0.00803518 | 0.03989126 | ANKRD35 |
| ENSG00000174501 | 1.75376139 | 1.62E-11 | 7.22E-10 | ANKRD36C |
| ENSG00000197043 | 4.94743696 | 0.00068621 | 0.00529332 | ANXA6 |
| ENSG00000248409 | -1.9249227 | 0.00172558 | 0.01144927 | AP000344.2 |
| ENSG00000107282 | 1.79349104 | 0.00019135 | 0.00176888 | APBA1 |
| ENSG00000184730 | -2.5682192 | 0.0044667 | 0.02480442 | APOBR |
| ENSG00000198576 | 6.78969705 | 5.23E-07 | 9.80E-06 | ARC |
| ENSG00000118520 | -2.3143356 | 0.00486632 | 0.0265303 | ARG1 |
| ENSG00000138639 | 1.57034454 | 0.00344835 | 0.02014394 | ARHGAP24 |
| ENSG00000089820 | -1.6893875 | 5.04E-14 | 3.48E-12 | ARHGAP4 |
| ENSG00000236699 | -2.279286 | 0.00472999 | 0.02594231 | ARHGEF38 |
| ENSG00000134108 | 1.60170861 | 8.34E-61 | 2.53E-57 | ARL8B |
| ENSG00000117407 | -2.408777 | 3.00E-06 | 4.66E-05 | ARTN |
| ENSG00000141505 | -2.3564993 | 3.85E-18 | 4.47E-16 | ASGR1 |
| ENSG00000108381 | -1.6685627 | 0.00076833 | 0.00582802 | ASPA |
| ENSG00000204653 | -2.1230192 | 0.00050533 | 0.00408956 | ASPDH |
| ENSG00000272899 | -1.7888569 | 0.00032166 | 0.00276319 | ATP6V1FNB |
| ENSG00000151418 | -3.5586102 | 0.00323459 | 0.01913648 | ATP6V1G3 |
| ENSG00000179766 | 2.4256783 | 5.61E-08 | 1.26E-06 | ATP8B5P |
| ENSG00000107518 | -2.8344746 | 1.68E-05 | 0.00021119 | ATRNL1 |
| ENSG00000166148 | -2.3886208 | 4.81E-17 | 4.98E-15 | AVPR1A |
| ENSG00000156966 | 2.87601415 | 2.29E-05 | 0.00027952 | B3GNT7 |
| ENSG00000237172 | -1.7476544 | 1.84E-13 | 1.16E-11 | B3GNT9 |
| ENSG00000129151 | -1.637893 | 3.12E-15 | 2.58E-13 | BBOX1 |
| ENSG00000060982 | -1.5871501 | 1.37E-09 | 4.25E-08 | BCAT1 |
| ENSG00000114200 | -2.0909947 | 4.09E-05 | 0.00046441 | BCHE |
| ENSG00000130303 | -3.7157744 | 2.47E-09 | 7.37E-08 | BST2 |
| ENSG00000113303 | -3.3433121 | 0.00011714 | 0.0011555 | BTNL8 |
| ENSG00000278704 | 3.06390745 | 0.0001134 | 0.00112356 | BX004987.1 |
| ENSG00000227051 | 1.8840113 | 0.00015345 | 0.00146073 | C14orf132 |
| ENSG00000184601 | -2.901434 | 0.00870966 | 0.04248545 | C14orf180 |
| ENSG00000278505 | -3.4270646 | 6.11E-07 | 1.12E-05 | C17orf78 |
| ENSG00000214212 | -3.169319 | 0.00012613 | 0.00123487 | C19orf38 |
| ENSG00000183186 | -3.5746419 | 0.00264016 | 0.01622931 | C2CD4C |
| ENSG00000204128 | -1.5866019 | 2.27E-23 | 4.81E-21 | C2orf72 |
| ENSG00000039537 | -2.0793067 | 1.02E-08 | 2.74E-07 | C6 |
| ENSG00000189325 | -2.3161842 | 6.78E-07 | 1.23E-05 | C6orf222 |
| ENSG00000185015 | -2.2285967 | 1.22E-05 | 0.00016221 | CA13 |
| ENSG00000158966 | 3.99140167 | 1.75E-18 | 2.07E-16 | CACHD1 |
| ENSG00000157445 | -2.7778067 | 8.48E-05 | 0.00087546 | CACNA2D3 |
| ENSG00000075461 | 3.49083618 | 4.40E-05 | 0.00049651 | CACNG4 |
| ENSG00000104327 | -3.4869134 | 7.65E-06 | 0.00010706 | CALB1 |
| ENSG00000172137 | 2.48813218 | 3.77E-47 | 5.29E-44 | CALB2 |
| ENSG00000077274 | -2.8693925 | 2.77E-39 | 2.30E-36 | CAPN6 |
| ENSG00000204397 | -1.5136601 | 0.0017023 | 0.01134449 | CARD16 |
| ENSG00000137752 | -2.3209813 | 1.06E-05 | 0.00014365 | CASP1 |
| ENSG00000142273 | -1.8321411 | 8.82E-13 | 4.94E-11 | CBLC |
| ENSG00000260220 | -1.6758943 | 0.00038942 | 0.00327417 | CCDC187 |
| ENSG00000236383 | -1.6803294 | 0.0033518 | 0.01967706 | CCDC200 |
| ENSG00000165972 | 2.94015737 | 0.00185154 | 0.01214884 | CCDC38 |
| ENSG00000198624 | -1.6335645 | 0.00082804 | 0.00621623 | CCDC69 |
| ENSG00000108691 | -6.1284304 | 4.90E-05 | 0.00054469 | CCL2 |
| ENSG00000163823 | -2.1331029 | 0.00607184 | 0.03173519 | CCR1 |
| ENSG00000249958 | -3.8481992 | 0.00460749 | 0.02547329 | CCT7P2 |
| ENSG00000122674 | 1.51988034 | 4.03E-10 | 1.37E-08 | CCZ1 |
| ENSG00000012124 | -1.9302064 | 1.21E-11 | 5.54E-10 | CD22 |
| ENSG00000122223 | 2.50096206 | 0.00619903 | 0.0322704 | CD244 |
| ENSG00000135218 | -3.1079566 | 1.25E-09 | 3.93E-08 | CD36 |
| ENSG00000019582 | -2.2461701 | 8.82E-08 | 1.92E-06 | CD74 |
| ENSG00000105369 | -2.345738 | 2.60E-09 | 7.74E-08 | CD79A |
| ENSG00000167617 | -3.1690499 | 1.46E-05 | 0.00018913 | CDC42EP5 |
| ENSG00000113361 | -4.1344845 | 9.19E-15 | 6.98E-13 | CDH6 |
| ENSG00000166446 | -1.7619118 | 9.24E-09 | 2.50E-07 | CDYL2 |
| ENSG00000171310 | 1.55724124 | 0.00024229 | 0.00216611 | CHST11 |
| ENSG00000135702 | -6.3067458 | 2.09E-06 | 3.38E-05 | CHST5 |
| ENSG00000136425 | 2.02485786 | 6.30E-10 | 2.08E-08 | CIB2 |
| ENSG00000066405 | -3.0537423 | 4.22E-13 | 2.54E-11 | CLDN18 |
| ENSG00000157335 | -2.3723271 | 0.00437448 | 0.02439637 | CLEC18C |
| ENSG00000236279 | 6.10102332 | 3.34E-05 | 0.00038777 | CLEC2L |
| ENSG00000166250 | 3.18957939 | 5.65E-30 | 2.15E-27 | CLMP |
| ENSG00000198515 | -2.2514293 | 0.00473572 | 0.02596596 | CNGA1 |
| ENSG00000142675 | 4.76014079 | 4.04E-08 | 9.31E-07 | CNKSR1 |
| ENSG00000106078 | 6.68893299 | 4.00E-60 | 1.04E-56 | COBL |
| ENSG00000197467 | 6.59310132 | 1.85E-06 | 3.02E-05 | COL13A1 |
| ENSG00000065618 | -1.7401588 | 1.02E-36 | 7.41E-34 | COL17A1 |
| ENSG00000108821 | -1.7289723 | 2.64E-05 | 0.00031694 | COL1A1 |
| ENSG00000171502 | -1.7308649 | 3.07E-13 | 1.89E-11 | COL24A1 |
| ENSG00000168542 | -5.9748574 | 1.47E-10 | 5.57E-09 | COL3A1 |
| ENSG00000080573 | -2.320166 | 1.26E-30 | 4.89E-28 | COL5A3 |
| ENSG00000114270 | 1.54349136 | 7.01E-08 | 1.55E-06 | COL7A1 |
| ENSG00000049089 | -2.0124897 | 1.24E-08 | 3.24E-07 | COL9A2 |
| ENSG00000196167 | -1.7276065 | 1.27E-25 | 3.21E-23 | COLCA1 |
| ENSG00000198756 | -4.9367003 | 5.75E-05 | 0.00062651 | COLGALT2 |
| ENSG00000103647 | 3.90970643 | 4.01E-08 | 9.26E-07 | CORO2B |
| ENSG00000165078 | -2.1577967 | 8.06E-11 | 3.19E-09 | CPA6 |
| ENSG00000106034 | -2.3834207 | 1.60E-13 | 1.03E-11 | CPED1 |
| ENSG00000135678 | 3.19958946 | 1.61E-17 | 1.75E-15 | CPM |
| ENSG00000178772 | -2.7824619 | 0.00598441 | 0.03135922 | CPN2 |
| ENSG00000140848 | 1.52267257 | 1.21E-12 | 6.45E-11 | CPNE2 |
| ENSG00000124772 | -1.6105529 | 0.01019768 | 0.04819758 | CPNE5 |
| ENSG00000104324 | -3.0218888 | 1.51E-34 | 8.90E-32 | CPQ |
| ENSG00000169509 | 6.31212492 | 9.59E-06 | 0.0001313 | CRCT1 |
| ENSG00000157613 | -3.4778572 | 5.74E-48 | 8.71E-45 | CREB3L1 |
| ENSG00000146592 | 3.35876217 | 3.37E-22 | 6.40E-20 | CREB5 |
| ENSG00000176092 | -1.5536512 | 8.67E-08 | 1.89E-06 | CRYBG2 |
| ENSG00000198930 | 5.18146407 | 1.30E-05 | 0.00017109 | CSAG1 |
| ENSG00000268916 | 7.10140238 | 8.21E-11 | 3.24E-09 | CSAG3 |
| ENSG00000172346 | -2.6006182 | 0.01042526 | 0.04901888 | CSDC2 |
| ENSG00000183117 | 5.90271673 | 7.06E-05 | 0.00074933 | CSMD1 |
| ENSG00000178662 | 2.10465847 | 0.00599014 | 0.03136216 | CSRNP3 |
| ENSG00000170373 | -3.1175898 | 8.01E-06 | 0.00011151 | CST1 |
| ENSG00000169551 | 9.01772271 | 1.69E-13 | 1.07E-11 | CT55 |
| ENSG00000126890 | 4.26850319 | 0.00223131 | 0.01414679 | CTAG2 |
| ENSG00000164932 | -2.9551456 | 3.13E-19 | 4.19E-17 | CTHRC1 |
| ENSG00000107611 | -2.3047916 | 0.00026725 | 0.00235684 | CUBN |
| ENSG00000165168 | 1.55620528 | 0.00085502 | 0.00638198 | CYBB |
| ENSG00000138061 | 1.95784823 | 5.01E-14 | 3.47E-12 | CYP1B1 |
| ENSG00000256612 | 1.90654883 | 2.64E-07 | 5.24E-06 | CYP2B7P |
| ENSG00000165841 | -2.6435857 | 0.00720118 | 0.03643586 | CYP2C19 |
| ENSG00000146233 | -1.8614154 | 0.00275408 | 0.01679433 | CYP39A1 |
| ENSG00000186115 | -1.7471211 | 0.00421244 | 0.02367388 | CYP4F2 |
| ENSG00000115165 | -2.2402261 | 0.00011013 | 0.00109531 | CYTIP |
| ENSG00000164488 | 2.66866002 | 5.31E-13 | 3.15E-11 | DACT2 |
| ENSG00000146038 | -1.9846423 | 1.74E-31 | 7.30E-29 | DCDC2 |
| ENSG00000077279 | -2.7176016 | 4.66E-05 | 0.0005207 | DCX |
| ENSG00000175984 | -1.6392517 | 1.39E-10 | 5.28E-09 | DENND2C |
| ENSG00000136267 | -1.7866118 | 0.00022326 | 0.00201896 | DGKB |
| ENSG00000174844 | -1.5142874 | 1.34E-05 | 0.00017558 | DNAH12 |
| ENSG00000272636 | 3.03901626 | 7.23E-09 | 2.00E-07 | DOC2B |
| ENSG00000245750 | -2.6032618 | 1.38E-07 | 2.89E-06 | DRAIC |
| ENSG00000149599 | -1.8560509 | 0.00363409 | 0.02103336 | DUSP15 |
| ENSG00000224807 | -2.7192663 | 0.00871691 | 0.04249991 | DUX4L9 |
| ENSG00000231513 | -3.6714302 | 0.00926443 | 0.04456124 | E2F6P4 |
| ENSG00000088881 | 1.72014076 | 1.34E-07 | 2.82E-06 | EBF4 |
| ENSG00000134463 | 3.56178745 | 1.61E-18 | 1.92E-16 | ECHDC3 |
| ENSG00000134109 | 1.86735482 | 1.50E-69 | 6.83E-66 | EDEM1 |
| ENSG00000127129 | -2.4521753 | 0.00431352 | 0.0241229 | EDN2 |
| ENSG00000115380 | 2.6792807 | 0.00102243 | 0.00743064 | EFEMP1 |
| ENSG00000163576 | -1.7916109 | 0.00314973 | 0.01873593 | EFHB |
| ENSG00000183690 | -3.3319095 | 0.00867196 | 0.04234101 | EFHC2 |
| ENSG00000084710 | 1.77243586 | 0.00400523 | 0.02273374 | EFR3B |
| ENSG00000172889 | 2.10762247 | 9.28E-10 | 2.97E-08 | EGFL7 |
| ENSG00000224057 | -2.9654052 | 3.92E-16 | 3.52E-14 | EGFR-AS1 |
| ENSG00000232415 | -2.1655586 | 0.00150962 | 0.01024401 | ELN-AS1 |
| ENSG00000165521 | -1.9177901 | 0.0019063 | 0.01244901 | EML5 |
| ENSG00000183317 | 1.69905979 | 0.0036115 | 0.02093592 | EPHA10 |
| ENSG00000154928 | -1.6293073 | 0.00736874 | 0.03711875 | EPHB1 |
| ENSG00000204978 | -3.5688281 | 3.83E-11 | 1.60E-09 | ERICH4 |
| ENSG00000086619 | 2.97571823 | 7.68E-17 | 7.86E-15 | ERO1B |
| ENSG00000106038 | -1.7786415 | 1.72E-05 | 0.00021547 | EVX1 |
| ENSG00000121769 | 1.74546018 | 6.95E-05 | 0.000741 | FABP3 |
| ENSG00000150510 | -1.5047841 | 0.00057355 | 0.00455146 | FAM124A |
| ENSG00000135842 | 1.93272366 | 0.00010845 | 0.00108156 | FAM129A |
| ENSG00000148541 | -2.4167003 | 0.00351941 | 0.02048679 | FAM13C |
| ENSG00000154319 | 2.03406573 | 4.39E-05 | 0.00049514 | FAM167A |
| ENSG00000164125 | -2.7454016 | 4.47E-15 | 3.60E-13 | FAM198B |
| ENSG00000108950 | 2.25763682 | 4.48E-09 | 1.28E-07 | FAM20A |
| ENSG00000177706 | 4.64179865 | 1.17E-23 | 2.58E-21 | FAM20C |
| ENSG00000224566 | -2.1678444 | 0.00442919 | 0.02464115 | FAM96AP2 |
| ENSG00000197601 | 3.19249269 | 0.009907 | 0.04709797 | FAR1 |
| ENSG00000026103 | 1.59666501 | 0.00051575 | 0.00415608 | FAS |
| ENSG00000196159 | -3.6047585 | 1.06E-09 | 3.38E-08 | FAT4 |
| ENSG00000163520 | 2.30351201 | 0.00365092 | 0.021104 | FBLN2 |
| ENSG00000183580 | -4.5977307 | 0.00025157 | 0.00223043 | FBXL7 |
| ENSG00000116661 | -1.9135392 | 3.75E-11 | 1.57E-09 | FBXO2 |
| ENSG00000230316 | -1.967076 | 0.00130543 | 0.00908514 | FEZF1-AS1 |
| ENSG00000113578 | -1.7715012 | 0.00014123 | 0.00135933 | FGF1 |
| ENSG00000129682 | -1.9292016 | 3.91E-18 | 4.51E-16 | FGF13 |
| ENSG00000171557 | -2.1144729 | 2.50E-35 | 1.57E-32 | FGG |
| ENSG00000176826 | 1.59069293 | 0.00112416 | 0.00804801 | FKBP9P1 |
| ENSG00000232774 | -2.5796734 | 6.54E-05 | 0.00069961 | FLJ22447 |
| ENSG00000155816 | -1.6022366 | 0.00035844 | 0.00304461 | FMN2 |
| ENSG00000007933 | -2.4549452 | 1.75E-17 | 1.90E-15 | FMO3 |
| ENSG00000251493 | 4.53163051 | 6.55E-08 | 1.45E-06 | FOXD1 |
| ENSG00000129654 | -2.451205 | 0.00276482 | 0.01684434 | FOXJ1 |
| ENSG00000179772 | -1.6270698 | 2.77E-09 | 8.21E-08 | FOXS1 |
| ENSG00000168843 | 2.0350637 | 9.91E-20 | 1.38E-17 | FSTL5 |
| ENSG00000176920 | -2.2536278 | 1.20E-23 | 2.60E-21 | FUT2 |
| ENSG00000156413 | -1.6526332 | 0.0007444 | 0.00566543 | FUT6 |
| ENSG00000110328 | 1.52115751 | 1.31E-06 | 2.24E-05 | GALNT18 |
| ENSG00000179348 | -2.2848337 | 5.97E-21 | 9.81E-19 | GATA2 |
| ENSG00000244300 | -1.7954304 | 1.94E-19 | 2.68E-17 | GATA2-AS1 |
| ENSG00000136574 | -1.7799349 | 1.00E-11 | 4.67E-10 | GATA4 |
| ENSG00000266010 | -1.6768362 | 2.70E-05 | 0.00032317 | GATA6-AS1 |
| ENSG00000145321 | -3.3609645 | 0.00538917 | 0.02879491 | GC |
| ENSG00000187210 | -1.6650298 | 1.23E-09 | 3.90E-08 | GCNT1 |
| ENSG00000130055 | -1.8484446 | 9.85E-07 | 1.73E-05 | GDPD2 |
| ENSG00000131459 | 2.50351191 | 1.84E-22 | 3.56E-20 | GFPT2 |
| ENSG00000152661 | 3.42084674 | 7.79E-16 | 6.69E-14 | GJA1 |
| ENSG00000169562 | -2.6351044 | 5.67E-11 | 2.31E-09 | GJB1 |
| ENSG00000101958 | -7.570597 | 5.96E-09 | 1.66E-07 | GLRA2 |
| ENSG00000260062 | -6.6687567 | 1.38E-06 | 2.34E-05 | GOLGA2P11 |
| ENSG00000143167 | -2.9643427 | 2.26E-09 | 6.78E-08 | GPA33 |
| ENSG00000183098 | 4.13213822 | 2.61E-45 | 2.80E-42 | GPC6 |
| ENSG00000119714 | -2.8427597 | 6.35E-06 | 9.07E-05 | GPR68 |
| ENSG00000185477 | 2.12828876 | 1.25E-08 | 3.26E-07 | GPRIN3 |
| ENSG00000125675 | -1.9003038 | 4.55E-07 | 8.70E-06 | GRIA3 |
| ENSG00000070019 | -3.7025877 | 2.13E-09 | 6.43E-08 | GUCY2C |
| ENSG00000162882 | 3.50457536 | 3.16E-24 | 7.11E-22 | HAAO |
| ENSG00000101323 | -3.1746942 | 1.79E-17 | 1.93E-15 | HAO1 |
| ENSG00000227214 | 3.22783447 | 0.00066995 | 0.00519202 | HCG15 |
| ENSG00000225914 | -2.2368218 | 0.00322197 | 0.01907223 | HCG23 |
| ENSG00000206337 | -2.827756 | 0.00045771 | 0.0037565 | HCP5 |
| ENSG00000089472 | -5.9340155 | 2.40E-08 | 5.88E-07 | HEPH |
| ENSG00000019991 | -2.648732 | 4.36E-16 | 3.88E-14 | HGF |
| ENSG00000187837 | -2.1666051 | 3.27E-54 | 6.62E-51 | HIST1H1C |
| ENSG00000180573 | -1.6176818 | 5.54E-19 | 7.06E-17 | HIST1H2AC |
| ENSG00000196747 | -3.9359771 | 0.00402018 | 0.0228044 | HIST1H2AI |
| ENSG00000180596 | -1.5974443 | 2.14E-07 | 4.32E-06 | HIST1H2BC |
| ENSG00000273802 | -1.5208823 | 4.56E-05 | 0.00051088 | HIST1H2BG |
| ENSG00000124635 | -2.2079996 | 6.88E-07 | 1.25E-05 | HIST1H2BJ |
| ENSG00000197903 | -1.824939 | 4.60E-36 | 2.99E-33 | HIST1H2BK |
| ENSG00000278828 | -2.0854152 | 4.81E-07 | 9.11E-06 | HIST1H3H |
| ENSG00000203812 | -3.272511 | 0.00227224 | 0.01436506 | HIST2H2AA3 |
| ENSG00000203814 | -1.6520202 | 1.13E-08 | 2.98E-07 | HIST2H2BF |
| ENSG00000183598 | -2.2048816 | 0.00634062 | 0.03283856 | HIST2H3D |
| ENSG00000270882 | -3.7529051 | 0.00276826 | 0.01684736 | HIST2H4A |
| ENSG00000270276 | -3.6953899 | 0.00232772 | 0.01463962 | HIST2H4B |
| ENSG00000159399 | -2.5515748 | 8.75E-05 | 0.00089916 | HK2 |
| ENSG00000234745 | -1.7378693 | 1.47E-22 | 2.92E-20 | HLA-B |
| ENSG00000204642 | -1.7169679 | 3.51E-05 | 0.00040607 | HLA-F |
| ENSG00000108924 | -1.8600819 | 0.00589077 | 0.03094862 | HLF |
| ENSG00000143341 | -3.314318 | 1.90E-53 | 3.46E-50 | HMCN1 |
| ENSG00000213559 | -5.8508823 | 9.01E-05 | 0.00092222 | HNRNPA1P64 |
| ENSG00000005073 | -2.5783603 | 3.84E-28 | 1.21E-25 | HOXA11 |
| ENSG00000240990 | -3.0889236 | 2.80E-12 | 1.42E-10 | HOXA11-AS |
| ENSG00000133328 | -1.700077 | 5.60E-12 | 2.71E-10 | HRASLS2 |
| ENSG00000167733 | -1.5765599 | 0.00025978 | 0.00229657 | HSD11B1L |
| ENSG00000176387 | -1.6425511 | 8.42E-11 | 3.29E-09 | HSD11B2 |
| ENSG00000086696 | -1.6150174 | 3.61E-12 | 1.79E-10 | HSD17B2 |
| ENSG00000025423 | -1.505314 | 0.00028387 | 0.002483 | HSD17B6 |
| ENSG00000135914 | -2.8048572 | 0.00572638 | 0.03021344 | HTR2B |
| ENSG00000170801 | 2.85040183 | 6.17E-22 | 1.09E-19 | HTRA3 |
| ENSG00000172201 | 4.12990271 | 0.00339958 | 0.01992289 | ID4 |
| ENSG00000103742 | 1.65950356 | 2.54E-06 | 4.01E-05 | IGDCC4 |
| ENSG00000115461 | 1.92536893 | 2.87E-31 | 1.16E-28 | IGFBP5 |
| ENSG00000204866 | -1.984546 | 6.76E-10 | 2.21E-08 | IGFL2 |
| ENSG00000144847 | -4.4665172 | 0.00049664 | 0.00403422 | IGSF11 |
| ENSG00000163501 | -2.2315043 | 4.55E-28 | 1.41E-25 | IHH |
| ENSG00000096996 | -1.7126855 | 0.00601255 | 0.03145236 | IL12RB1 |
| ENSG00000123496 | -1.9528444 | 4.25E-81 | 7.75E-77 | IL13RA2 |
| ENSG00000188263 | -1.9977219 | 0.00132381 | 0.00918151 | IL17REL |
| ENSG00000168685 | 1.58819068 | 9.82E-10 | 3.13E-08 | IL7R |
| ENSG00000122641 | 2.31082001 | 0.00591057 | 0.03102579 | INHBA |
| ENSG00000168918 | -5.9444089 | 5.91E-05 | 0.00064043 | INPP5D |
| ENSG00000130518 | -2.2670424 | 2.09E-09 | 6.34E-08 | IQCN |
| ENSG00000140678 | 2.29046553 | 1.13E-09 | 3.59E-08 | ITGAX |
| ENSG00000179914 | 3.39047967 | 0.0001073 | 0.00107296 | ITLN1 |
| ENSG00000099840 | -1.6393151 | 1.48E-05 | 0.00019093 | IZUMO4 |
| ENSG00000280780 | -4.0700859 | 0.0022664 | 0.01434311 | JAKMIP2-AS1 |
| ENSG00000160593 | -1.7988755 | 2.18E-09 | 6.57E-08 | JAML |
| ENSG00000175538 | 3.13989701 | 4.06E-13 | 2.46E-11 | KCNE3 |
| ENSG00000187486 | -1.6962764 | 0.00520389 | 0.02801859 | KCNJ11 |
| ENSG00000153822 | 2.32641052 | 5.50E-05 | 0.00060236 | KCNJ16 |
| ENSG00000184156 | 1.64418674 | 1.69E-20 | 2.63E-18 | KCNQ3 |
| ENSG00000175707 | -1.8150129 | 4.10E-06 | 6.15E-05 | KDF1 |
| ENSG00000128052 | 4.94256276 | 1.53E-10 | 5.75E-09 | KDR |
| ENSG00000110427 | 5.43252689 | 4.17E-15 | 3.39E-13 | KIAA1549L |
| ENSG00000162849 | -2.4367832 | 5.07E-14 | 3.49E-12 | KIF26B |
| ENSG00000134962 | -3.7744574 | 1.29E-09 | 4.04E-08 | KLB |
| ENSG00000133619 | 2.26399641 | 2.21E-15 | 1.86E-13 | KRBA1 |
| ENSG00000227300 | -3.2669698 | 2.17E-08 | 5.38E-07 | KRT16P2 |
| ENSG00000226145 | -4.7474457 | 0.00159874 | 0.01074871 | KRT16P6 |
| ENSG00000128422 | -1.8067211 | 0.00044924 | 0.00369703 | KRT17 |
| ENSG00000108244 | -2.6782665 | 1.82E-05 | 0.00022651 | KRT23 |
| ENSG00000135480 | 2.53628211 | 1.44E-32 | 6.73E-30 | KRT7 |
| ENSG00000248807 | 3.0919232 | 0.00466002 | 0.02568228 | KRTAP9-12P |
| ENSG00000198910 | 1.63848552 | 5.56E-06 | 8.03E-05 | L1CAM |
| ENSG00000154655 | -5.2688861 | 1.01E-07 | 2.17E-06 | L3MBTL4 |
| ENSG00000184925 | -2.5100816 | 0.00850128 | 0.04169402 | LCN12 |
| ENSG00000136167 | 1.85239871 | 7.13E-32 | 3.17E-29 | LCP1 |
| ENSG00000166816 | -1.8574277 | 5.47E-05 | 0.00059929 | LDHD |
| ENSG00000203985 | -1.9008715 | 0.00142535 | 0.00977411 | LDLRAD1 |
| ENSG00000168675 | -2.0216945 | 0.00371532 | 0.02141513 | LDLRAD4 |
| ENSG00000170298 | -2.5058546 | 0.00249116 | 0.01552702 | LGALS9B |
| ENSG00000139292 | -8.0121821 | 6.34E-11 | 2.57E-09 | LGR5 |
| ENSG00000187416 | -2.2772864 | 0.00433908 | 0.02422123 | LHFPL3 |
| ENSG00000225329 | -1.6936577 | 0.00506893 | 0.0274216 | LHFPL3-AS2 |
| ENSG00000250682 | 3.37688628 | 0.00463875 | 0.02557278 | LINC00491 |
| ENSG00000276476 | 6.90587923 | 2.46E-07 | 4.91E-06 | LINC00540 |
| ENSG00000259070 | -3.8198134 | 0.00013676 | 0.00132077 | LINC00639 |
| ENSG00000259129 | -2.4999011 | 3.10E-10 | 1.09E-08 | LINC00648 |
| ENSG00000247982 | -1.6661332 | 6.12E-06 | 8.76E-05 | LINC00926 |
| ENSG00000248698 | -2.197148 | 0.00189316 | 0.01237209 | LINC01085 |
| ENSG00000224259 | -1.8432278 | 1.36E-16 | 1.34E-14 | LINC01133 |
| ENSG00000258867 | 1.81971965 | 4.55E-07 | 8.71E-06 | LINC01146 |
| ENSG00000259471 | -3.6572123 | 0.00147608 | 0.01004264 | LINC01169 |
| ENSG00000284543 | -1.8440831 | 0.00097225 | 0.00712854 | LINC01226 |
| ENSG00000227953 | -1.7665414 | 0.001558 | 0.0105175 | LINC01341 |
| ENSG00000246223 | 2.76207123 | 0.00670671 | 0.03438275 | LINC01550 |
| ENSG00000231776 | -2.0764007 | 0.00266708 | 0.01634969 | LINC01611 |
| ENSG00000234184 | -7.123112 | 9.99E-08 | 2.16E-06 | LINC01781 |
| ENSG00000259439 | 2.36088933 | 2.28E-10 | 8.21E-09 | LINC01833 |
| ENSG00000266258 | -1.8257597 | 0.0003982 | 0.00333266 | LINC01909 |
| ENSG00000267013 | -3.7375918 | 1.46E-07 | 3.04E-06 | LINC01929 |
| ENSG00000254101 | 6.94674597 | 2.17E-07 | 4.39E-06 | LINC02055 |
| ENSG00000261175 | 2.91823872 | 3.21E-08 | 7.64E-07 | LINC02188 |
| ENSG00000281903 | 4.3282231 | 5.72E-14 | 3.91E-12 | LINC02246 |
| ENSG00000256643 | 2.19011216 | 2.69E-08 | 6.54E-07 | LINC02441 |
| ENSG00000248869 | -2.62099 | 0.0010548 | 0.00763852 | LINC02511 |
| ENSG00000261780 | -5.9124882 | 0.00013398 | 0.00129844 | LINC02582 |
| ENSG00000166035 | -1.5573443 | 5.53E-13 | 3.27E-11 | LIPC |
| ENSG00000079435 | -3.074918 | 9.69E-06 | 0.00013243 | LIPE |
| ENSG00000138131 | -2.2422604 | 4.35E-22 | 8.01E-20 | LOXL4 |
| ENSG00000168702 | -5.7560266 | 2.24E-17 | 2.38E-15 | LRP1B |
| ENSG00000188993 | -1.5778208 | 0.00193183 | 0.01258721 | LRRC66 |
| ENSG00000160932 | 1.65414721 | 1.29E-25 | 3.21E-23 | LY6E |
| ENSG00000090382 | -3.6455488 | 6.48E-33 | 3.19E-30 | LYZ |
| ENSG00000181541 | -4.6868843 | 0.00201603 | 0.01307655 | MAB21L2 |
| ENSG00000183742 | -1.7488805 | 0.00036742 | 0.00310991 | MACC1 |
| ENSG00000185247 | 8.08367228 | 2.88E-10 | 1.02E-08 | MAGEA11 |
| ENSG00000213401 | 5.917593 | 1.45E-07 | 3.03E-06 | MAGEA12 |
| ENSG00000197172 | 4.61762982 | 4.48E-20 | 6.79E-18 | MAGEA6 |
| ENSG00000099399 | 6.52883827 | 2.98E-06 | 4.63E-05 | MAGEB2 |
| ENSG00000198934 | 4.76062654 | 2.39E-11 | 1.04E-09 | MAGEE1 |
| ENSG00000204706 | -3.154582 | 0.00145971 | 0.00995725 | MAMDC2-AS1 |
| ENSG00000116141 | 4.68396958 | 0.00272177 | 0.01663559 | MARK1 |
| ENSG00000140832 | -2.9433685 | 1.03E-07 | 2.21E-06 | MARVELD3 |
| ENSG00000151224 | -4.3691436 | 4.01E-06 | 6.03E-05 | MAT1A |
| ENSG00000180611 | 1.55216794 | 0.00328854 | 0.01938538 | MB21D2 |
| ENSG00000076770 | -5.1000373 | 7.93E-23 | 1.64E-20 | MBNL3 |
| ENSG00000214548 | 1.999376 | 0.00124002 | 0.00870977 | MEG3 |
| ENSG00000112818 | -6.0509535 | 2.29E-21 | 3.83E-19 | MEP1A |
| ENSG00000182050 | 3.27563257 | 4.03E-07 | 7.80E-06 | MGAT4C |
| ENSG00000100253 | -1.7235391 | 0.00721512 | 0.03648611 | MIOX |
| ENSG00000224141 | -2.5104886 | 0.00066343 | 0.00514807 | MIR548XHG |
| ENSG00000187098 | 1.7869052 | 0.00026221 | 0.0023158 | MITF |
| ENSG00000196611 | -1.9876478 | 9.26E-52 | 1.53E-48 | MMP1 |
| ENSG00000137745 | -2.6714204 | 5.05E-07 | 9.52E-06 | MMP13 |
| ENSG00000271447 | -3.2769846 | 6.70E-21 | 1.08E-18 | MMP28 |
| ENSG00000106384 | -1.7039882 | 4.61E-11 | 1.91E-09 | MOGAT3 |
| ENSG00000079931 | 1.99987372 | 0.00012915 | 0.00126037 | MOXD1 |
| ENSG00000107186 | 8.12022871 | 1.02E-10 | 3.94E-09 | MPDZ |
| ENSG00000150054 | -1.7196455 | 4.31E-07 | 8.29E-06 | MPP7 |
| ENSG00000134042 | -3.4685707 | 0.00458327 | 0.025359 | MRO |
| ENSG00000185038 | -1.6025401 | 8.25E-07 | 1.48E-05 | MROH2A |
| ENSG00000166959 | -2.2816355 | 1.72E-11 | 7.62E-10 | MS4A8 |
| ENSG00000164078 | -2.0052604 | 3.84E-08 | 8.95E-07 | MST1R |
| ENSG00000169876 | -2.947307 | 1.70E-06 | 2.81E-05 | MUC17 |
| ENSG00000117983 | 1.7245981 | 9.85E-23 | 2.02E-20 | MUC5B |
| ENSG00000030304 | 2.83729261 | 8.53E-10 | 2.76E-08 | MUSK |
| ENSG00000196091 | -1.5611068 | 3.99E-08 | 9.25E-07 | MYBPC1 |
| ENSG00000134323 | 2.57629528 | 3.09E-06 | 4.78E-05 | MYCN |
| ENSG00000166866 | -1.7584216 | 2.30E-10 | 8.27E-09 | MYO1A |
| ENSG00000101605 | -2.3709205 | 3.33E-08 | 7.90E-07 | MYOM1 |
| ENSG00000139597 | -3.3310915 | 3.65E-06 | 5.55E-05 | N4BP2L1 |
| ENSG00000145911 | 3.97339555 | 3.36E-15 | 2.74E-13 | N4BP3 |
| ENSG00000156006 | -1.7038284 | 0.007371 | 0.03711983 | NAT2 |
| ENSG00000154654 | 6.13484706 | 2.08E-05 | 0.00025546 | NCAM2 |
| ENSG00000230257 | 1.99224124 | 3.18E-37 | 2.52E-34 | NFE4 |
| ENSG00000188158 | 1.82409685 | 7.28E-13 | 4.21E-11 | NHS |
| ENSG00000185942 | 3.50269229 | 5.28E-12 | 2.57E-10 | NKAIN3 |
| ENSG00000274956 | 8.78548022 | 8.32E-13 | 4.72E-11 | NKAIN3-IT1 |
| ENSG00000140807 | -2.3184497 | 7.75E-29 | 2.62E-26 | NKD1 |
| ENSG00000125820 | -4.7436024 | 1.47E-15 | 1.25E-13 | NKX2-2 |
| ENSG00000165246 | -3.4234958 | 1.19E-05 | 0.0001586 | NLGN4Y |
| ENSG00000091592 | 2.03445815 | 7.98E-11 | 3.16E-09 | NLRP1 |
| ENSG00000185269 | -2.8214067 | 6.94E-11 | 2.79E-09 | NOTUM |
| ENSG00000007952 | -1.5191687 | 5.47E-10 | 1.82E-08 | NOX1 |
| ENSG00000169418 | 1.91166626 | 0.00032717 | 0.00280519 | NPR1 |
| ENSG00000131910 | -6.3834314 | 5.69E-06 | 8.21E-05 | NR0B2 |
| ENSG00000180530 | -2.9668274 | 3.13E-09 | 9.22E-08 | NRIP1 |
| ENSG00000179915 | -3.1760737 | 0.00073803 | 0.00562629 | NRXN1 |
| ENSG00000269405 | 5.71052473 | 0.00024779 | 0.00220519 | NXF2 |
| ENSG00000183251 | -3.9230857 | 0.00054944 | 0.00437726 | OR51B4 |
| ENSG00000167355 | -3.0588854 | 5.71E-05 | 0.00062292 | OR51B5 |
| ENSG00000229314 | -1.6297158 | 0.001513 | 0.01025934 | ORM1 |
| ENSG00000162881 | 4.03739811 | 1.16E-08 | 3.04E-07 | OXER1 |
| ENSG00000180914 | 1.59702009 | 5.89E-05 | 0.00063964 | OXTR |
| ENSG00000169860 | 3.29703522 | 8.75E-14 | 5.86E-12 | P2RY1 |
| ENSG00000171759 | -3.9764178 | 7.35E-47 | 9.57E-44 | PAH |
| ENSG00000188582 | 2.30093505 | 9.44E-19 | 1.18E-16 | PAQR9 |
| ENSG00000241570 | 2.01782256 | 0.00614382 | 0.03202871 | PAQR9-AS1 |
| ENSG00000116117 | -2.6290844 | 2.96E-05 | 0.00035011 | PARD3B |
| ENSG00000151883 | -2.7097744 | 1.43E-18 | 1.72E-16 | PARP8 |
| ENSG00000007372 | -2.1537949 | 6.14E-05 | 0.00066259 | PAX6 |
| ENSG00000243232 | 6.09924642 | 3.94E-08 | 9.16E-07 | PCDHAC2 |
| ENSG00000197479 | -1.7957162 | 0.0017434 | 0.0115466 | PCDHB11 |
| ENSG00000253910 | 1.60438981 | 0.00098937 | 0.00722498 | PCDHGB2 |
| ENSG00000186472 | -2.3697254 | 2.27E-08 | 5.59E-07 | PCLO |
| ENSG00000102109 | 7.053541 | 1.08E-07 | 2.32E-06 | PCSK1N |
| ENSG00000128655 | -1.5216333 | 0.00050571 | 0.0040897 | PDE11A |
| ENSG00000172572 | -3.4717479 | 9.41E-13 | 5.25E-11 | PDE3A |
| ENSG00000160191 | 1.67529859 | 1.04E-06 | 1.81E-05 | PDE9A |
| ENSG00000145431 | 3.96429868 | 1.22E-21 | 2.13E-19 | PDGFC |
| ENSG00000163737 | -1.6374093 | 4.65E-05 | 0.00051978 | PF4 |
| ENSG00000092621 | 2.71256343 | 9.49E-46 | 1.08E-42 | PHGDH |
| ENSG00000233041 | -3.3793821 | 1.15E-05 | 0.00015454 | PHGR1 |
| ENSG00000174307 | 3.95375397 | 4.76E-15 | 3.82E-13 | PHLDA3 |
| ENSG00000165443 | -2.7454131 | 2.85E-36 | 1.92E-33 | PHYHIPL |
| ENSG00000137558 | -1.6194733 | 0.0051029 | 0.02756443 | PI15 |
| ENSG00000153823 | -2.7048169 | 4.66E-09 | 1.32E-07 | PID1 |
| ENSG00000162896 | -2.8211071 | 2.40E-06 | 3.81E-05 | PIGR |
| ENSG00000171033 | -6.1175829 | 3.74E-08 | 8.77E-07 | PKIA |
| ENSG00000254266 | -3.3880231 | 4.25E-05 | 0.0004807 | PKIA-AS1 |
| ENSG00000143627 | -2.3330549 | 1.69E-05 | 0.00021307 | PKLR |
| ENSG00000188257 | -2.719033 | 0.00013012 | 0.00126848 | PLA2G2A |
| ENSG00000145287 | -1.8160094 | 0.00031613 | 0.0027208 | PLAC8 |
| ENSG00000121316 | 2.68646186 | 1.28E-07 | 2.70E-06 | PLBD1 |
| ENSG00000021300 | 1.71481873 | 5.15E-06 | 7.55E-05 | PLEKHB1 |
| ENSG00000153404 | -3.2090421 | 4.12E-18 | 4.72E-16 | PLEKHG4B |
| ENSG00000166819 | -1.690539 | 0.00495835 | 0.02691928 | PLIN1 |
| ENSG00000130822 | -1.5123202 | 0.00137866 | 0.00951483 | PNCK |
| ENSG00000240694 | 2.16652766 | 1.58E-11 | 7.06E-10 | PNMA2 |
| ENSG00000128567 | 1.89731821 | 5.74E-07 | 1.06E-05 | PODXL |
| ENSG00000130997 | -2.6494999 | 0.00215323 | 0.01375108 | POLN |
| ENSG00000105852 | -1.9669408 | 2.25E-16 | 2.11E-14 | PON3 |
| ENSG00000110777 | -2.3457639 | 1.35E-18 | 1.64E-16 | POU2AF1 |
| ENSG00000163736 | -3.3982868 | 0.0063074 | 0.03270046 | PPBP |
| ENSG00000258116 | -3.6313624 | 1.82E-15 | 1.54E-13 | PPIAP45 |
| ENSG00000131771 | -2.291949 | 4.98E-06 | 7.34E-05 | PPP1R1B |
| ENSG00000219607 | -1.7220286 | 1.67E-05 | 0.00021074 | PPP1R3G |
| ENSG00000156475 | 4.33157204 | 3.42E-05 | 0.0003962 | PPP2R2B |
| ENSG00000224960 | -6.2198731 | 1.36E-05 | 0.00017778 | PPP4R3C |
| ENSG00000119698 | 3.80073351 | 3.80E-08 | 8.88E-07 | PPP4R4 |
| ENSG00000061455 | 2.30784556 | 0.00082469 | 0.00619621 | PRDM6 |
| ENSG00000164256 | -6.1133316 | 2.68E-05 | 0.00032083 | PRDM9 |
| ENSG00000166501 | 9.013952 | 6.16E-24 | 1.37E-21 | PRKCB |
| ENSG00000259205 | -1.7507684 | 0.00168144 | 0.01121372 | PRKXP1 |
| ENSG00000113494 | -1.6387675 | 9.28E-09 | 2.51E-07 | PRLR |
| ENSG00000100033 | -4.2263682 | 0.00855799 | 0.04191573 | PRODH |
| ENSG00000250799 | -1.53021 | 1.65E-13 | 1.06E-11 | PRODH2 |
| ENSG00000184500 | -1.551236 | 1.13E-17 | 1.26E-15 | PROS1 |
| ENSG00000167183 | -3.1085159 | 2.48E-11 | 1.08E-09 | PRR15L |
| ENSG00000212123 | -1.8026246 | 4.15E-09 | 1.19E-07 | PRR22 |
| ENSG00000164099 | 1.66694967 | 1.09E-26 | 2.97E-24 | PRSS12 |
| ENSG00000146250 | -2.8944112 | 2.06E-19 | 2.82E-17 | PRSS35 |
| ENSG00000052344 | -1.829112 | 0.00044629 | 0.003676 | PRSS8 |
| ENSG00000167653 | -1.8316316 | 0.00114843 | 0.00819601 | PSCA |
| ENSG00000081237 | -5.0429129 | 2.39E-22 | 4.59E-20 | PTPRC |
| ENSG00000060656 | 2.39451924 | 1.42E-09 | 4.40E-08 | PTPRU |
| ENSG00000250337 | -4.3522042 | 4.47E-05 | 0.00050257 | PURPL |
| ENSG00000101417 | -1.7609036 | 5.90E-16 | 5.14E-14 | PXMP4 |
| ENSG00000101074 | -2.1373106 | 9.67E-06 | 0.00013224 | R3HDML |
| ENSG00000154917 | 3.17112225 | 1.12E-08 | 2.95E-07 | RAB6B |
| ENSG00000106538 | -1.8494946 | 9.49E-13 | 5.26E-11 | RARRES2 |
| ENSG00000198915 | 4.91141371 | 0.00106147 | 0.00767155 | RASGEF1A |
| ENSG00000122035 | -2.3416671 | 1.19E-12 | 6.39E-11 | RASL11A |
| ENSG00000101265 | 4.20078693 | 1.59E-29 | 5.56E-27 | RASSF2 |
| ENSG00000266094 | 1.5352351 | 2.68E-07 | 5.31E-06 | RASSF5 |
| ENSG00000236718 | -1.6734482 | 0.00271952 | 0.01662741 | RBMY2QP |
| ENSG00000114115 | 3.98641126 | 0.00363156 | 0.02103212 | RBP1 |
| ENSG00000172348 | -3.4342961 | 1.00E-18 | 1.24E-16 | RCAN2 |
| ENSG00000134193 | -4.6106522 | 3.62E-12 | 1.79E-10 | REG4 |
| ENSG00000102032 | -1.9659018 | 2.92E-24 | 6.64E-22 | RENBP |
| ENSG00000154153 | -2.8275031 | 0.00122028 | 0.00860307 | RETREG1 |
| ENSG00000251258 | 6.86673022 | 7.04E-15 | 5.51E-13 | RFPL4B |
| ENSG00000185002 | -6.2015078 | 3.65E-06 | 5.55E-05 | RFX6 |
| ENSG00000102760 | 2.22280265 | 3.20E-08 | 7.64E-07 | RGCC |
| ENSG00000127074 | -2.2592806 | 0.00238226 | 0.01494142 | RGS13 |
| ENSG00000091844 | 1.5723675 | 4.33E-15 | 3.51E-13 | RGS17 |
| ENSG00000147509 | 1.85063117 | 4.74E-06 | 7.02E-05 | RGS20 |
| ENSG00000132554 | 1.7271054 | 0.00536746 | 0.02870925 | RGS22 |
| ENSG00000117152 | -1.8562143 | 0.00021791 | 0.00197721 | RGS4 |
| ENSG00000258545 | -2.9783313 | 5.93E-14 | 4.04E-12 | RHOXF1-AS1 |
| ENSG00000255794 | -2.3283398 | 9.99E-05 | 0.00100982 | RMST |
| ENSG00000278771 | -2.9742268 | 8.98E-06 | 0.00012364 | RN7SL3 |
| ENSG00000223318 | -2.2488267 | 0.00093803 | 0.00691096 | RNA5SP111 |
| ENSG00000129538 | -1.9831789 | 1.93E-12 | 9.97E-11 | RNASE1 |
| ENSG00000228203 | 2.86898113 | 0.00186418 | 0.01220896 | RNF144A-AS1 |
| ENSG00000178828 | -2.1371241 | 0.00064076 | 0.00499984 | RNF186 |
| ENSG00000143365 | -3.5788322 | 2.74E-16 | 2.52E-14 | RORC |
| ENSG00000243742 | -2.9671506 | 3.75E-05 | 0.00042952 | RPLP0P2 |
| ENSG00000189334 | -1.7189904 | 4.12E-05 | 0.00046802 | S100A14 |
| ENSG00000187634 | -2.0737277 | 0.00031338 | 0.00270221 | SAMD11 |
| ENSG00000182568 | -3.4887788 | 3.30E-06 | 5.06E-05 | SATB1 |
| ENSG00000145284 | 4.87008294 | 2.40E-15 | 2.00E-13 | SCD5 |
| ENSG00000079689 | -1.583108 | 0.00106424 | 0.00768849 | SCGN |
| ENSG00000136531 | 2.88832324 | 5.35E-06 | 7.78E-05 | SCN2A |
| ENSG00000184860 | 3.07548045 | 4.23E-10 | 1.42E-08 | SDR42E1 |
| ENSG00000010319 | -1.893727 | 3.93E-09 | 1.14E-07 | SEMA3G |
| ENSG00000100665 | 1.72857456 | 0.00610588 | 0.03186741 | SERPINA4 |
| ENSG00000188488 | 2.43831322 | 9.46E-12 | 4.43E-10 | SERPINA5 |
| ENSG00000170099 | 1.82306298 | 1.59E-09 | 4.89E-08 | SERPINA6 |
| ENSG00000206072 | -2.3443729 | 3.26E-10 | 1.14E-08 | SERPINB11 |
| ENSG00000163536 | -3.1843908 | 0.00470267 | 0.02582359 | SERPINI1 |
| ENSG00000149212 | -2.0296963 | 0.00065495 | 0.00509964 | SESN3 |
| ENSG00000162105 | -1.9813814 | 0.00347051 | 0.02024739 | SHANK2 |
| ENSG00000251322 | 2.77409347 | 1.05E-57 | 2.40E-54 | SHANK3 |
| ENSG00000129946 | -2.9542749 | 2.56E-77 | 2.33E-73 | SHC2 |
| ENSG00000148082 | -1.7225389 | 1.50E-07 | 3.10E-06 | SHC3 |
| ENSG00000105251 | -2.0133637 | 0.0012759 | 0.00892 | SHD |
| ENSG00000197046 | -2.1877146 | 2.75E-05 | 0.00032727 | SIGLEC15 |
| ENSG00000198053 | 1.60656699 | 7.17E-20 | 1.04E-17 | SIRPA |
| ENSG00000139737 | 6.26603458 | 1.42E-05 | 0.00018434 | SLAIN1 |
| ENSG00000026751 | 2.95858898 | 0.00043565 | 0.00359326 | SLAMF7 |
| ENSG00000110446 | -2.0383499 | 5.98E-09 | 1.67E-07 | SLC15A3 |
| ENSG00000179520 | -2.1167682 | 0.0007008 | 0.00538755 | SLC17A8 |
| ENSG00000156222 | -2.2915047 | 3.98E-06 | 6.00E-05 | SLC28A1 |
| ENSG00000197496 | 2.65338893 | 1.22E-06 | 2.11E-05 | SLC2A10 |
| ENSG00000182747 | 4.31767435 | 0.00071322 | 0.00546461 | SLC35D3 |
| ENSG00000169507 | -3.2917374 | 1.21E-42 | 1.10E-39 | SLC38A11 |
| ENSG00000139540 | -1.7721625 | 2.64E-07 | 5.24E-06 | SLC39A5 |
| ENSG00000134802 | 1.81084892 | 1.92E-21 | 3.32E-19 | SLC43A3 |
| ENSG00000129353 | -1.6647798 | 3.72E-13 | 2.27E-11 | SLC44A2 |
| ENSG00000204385 | -1.8256628 | 3.53E-06 | 5.39E-05 | SLC44A4 |
| ENSG00000076351 | 3.8280103 | 0.00479169 | 0.02620974 | SLC46A1 |
| ENSG00000111181 | -2.3049509 | 5.00E-09 | 1.41E-07 | SLC6A12 |
| ENSG00000268104 | 3.40533664 | 0.00112609 | 0.00805864 | SLC6A14 |
| ENSG00000108576 | -2.0465845 | 0.00607964 | 0.03176688 | SLC6A4 |
| ENSG00000092068 | -2.477855 | 1.83E-06 | 2.99E-05 | SLC7A8 |
| ENSG00000180251 | -2.7190627 | 4.94E-19 | 6.43E-17 | SLC9A4 |
| ENSG00000145147 | 2.37169056 | 6.43E-05 | 0.00069016 | SLIT2 |
| ENSG00000185710 | 1.65375513 | 0.00497814 | 0.02700261 | SMG1P4 |
| ENSG00000184785 | 7.54852598 | 3.99E-09 | 1.15E-07 | SMIM10 |
| ENSG00000256235 | 3.76824264 | 0.00614961 | 0.03204059 | SMIM3 |
| ENSG00000128602 | 1.74671084 | 4.99E-12 | 2.45E-10 | SMO |
| ENSG00000167941 | -4.1216848 | 0.00247758 | 0.01546206 | SOST |
| ENSG00000134532 | -6.0390344 | 3.76E-32 | 1.71E-29 | SOX5 |
| ENSG00000204335 | -3.3065915 | 4.69E-05 | 0.00052324 | SP5 |
| ENSG00000203926 | 3.92620848 | 0.00050988 | 0.00411791 | SPANXA2 |
| ENSG00000227234 | 2.90574293 | 3.47E-09 | 1.01E-07 | SPANXB1 |
| ENSG00000196406 | 3.95612389 | 1.27E-09 | 3.98E-08 | SPANXD |
| ENSG00000152583 | -2.7763504 | 3.45E-28 | 1.12E-25 | SPARCL1 |
| ENSG00000160284 | 2.65510506 | 0.00089012 | 0.00659267 | SPATC1L |
| ENSG00000134668 | -2.2096813 | 4.22E-22 | 7.85E-20 | SPOCD1 |
| ENSG00000137877 | -1.5654416 | 1.27E-12 | 6.75E-11 | SPTBN5 |
| ENSG00000101955 | 1.71293749 | 2.83E-12 | 1.42E-10 | SRPX |
| ENSG00000179954 | -2.5029797 | 0.00712372 | 0.03615439 | SSC5D |
| ENSG00000123096 | 2.4147508 | 3.93E-07 | 7.62E-06 | SSPN |
| ENSG00000144681 | 7.82509152 | 1.81E-10 | 6.66E-09 | STAC |
| ENSG00000178078 | -1.646495 | 1.76E-31 | 7.30E-29 | STAP2 |
| ENSG00000060140 | -2.337588 | 9.75E-06 | 0.00013307 | STYK1 |
| ENSG00000198829 | -1.6647523 | 0.00969289 | 0.0462583 | SUCNR1 |
| ENSG00000173597 | -3.0770926 | 1.09E-46 | 1.33E-43 | SULT1B1 |
| ENSG00000165124 | 3.24720631 | 0.00845666 | 0.04153455 | SVEP1 |
| ENSG00000181392 | -2.0803336 | 2.83E-10 | 1.00E-08 | SYNE4 |
| ENSG00000173227 | 1.68526141 | 2.85E-08 | 6.87E-07 | SYT12 |
| ENSG00000149043 | -1.5995545 | 1.61E-05 | 0.00020545 | SYT8 |
| ENSG00000146383 | -2.3764462 | 0.00402858 | 0.0228307 | TAAR6 |
| ENSG00000149591 | -1.7492909 | 1.08E-20 | 1.72E-18 | TAGLN |
| ENSG00000266733 | 2.31237539 | 0.00972446 | 0.04638325 | TBC1D29 |
| ENSG00000136111 | 1.62041488 | 9.67E-20 | 1.36E-17 | TBC1D4 |
| ENSG00000198933 | 1.62168227 | 0.00157412 | 0.01061061 | TBKBP1 |
| ENSG00000135111 | -1.864875 | 6.96E-30 | 2.59E-27 | TBX3 |
| ENSG00000149922 | -1.5098247 | 0.00011432 | 0.00113012 | TBX6 |
| ENSG00000152284 | 3.55437345 | 1.50E-18 | 1.80E-16 | TCF7L1 |
| ENSG00000188681 | -1.9683036 | 3.25E-06 | 5.00E-05 | TEKT4P2 |
| ENSG00000183508 | -1.8665951 | 0.00025022 | 0.00222278 | TENT5C |
| ENSG00000091513 | 1.70035712 | 7.71E-21 | 1.23E-18 | TF |
| ENSG00000115112 | 4.07339555 | 1.01E-12 | 5.52E-11 | TFCP2L1 |
| ENSG00000160182 | -2.5324791 | 2.48E-06 | 3.94E-05 | TFF1 |
| ENSG00000160181 | -2.5491312 | 1.12E-29 | 4.09E-27 | TFF2 |
| ENSG00000232480 | -1.7635598 | 0.001506 | 0.01022713 | TGFB2-AS1 |
| ENSG00000144115 | 6.80622382 | 1.49E-31 | 6.44E-29 | THNSL2 |
| ENSG00000106829 | -2.1123202 | 1.39E-13 | 9.05E-12 | TLE4 |
| ENSG00000104953 | -2.5691543 | 0.00860327 | 0.0420993 | TLE6 |
| ENSG00000133069 | 1.51025288 | 0.00463386 | 0.02556288 | TMCC2 |
| ENSG00000157315 | -1.9073582 | 0.00325285 | 0.01921753 | TMED6 |
| ENSG00000180061 | -1.7309818 | 4.09E-10 | 1.38E-08 | TMEM150B |
| ENSG00000152128 | 3.32269381 | 1.49E-42 | 1.29E-39 | TMEM163 |
| ENSG00000188760 | -2.342168 | 7.61E-09 | 2.09E-07 | TMEM198 |
| ENSG00000206432 | -6.3761871 | 7.26E-06 | 0.00010254 | TMEM200C |
| ENSG00000205084 | -1.9256262 | 0.00380049 | 0.02180256 | TMEM231 |
| ENSG00000165152 | 1.64041718 | 0.00475509 | 0.0260408 | TMEM246 |
| ENSG00000149582 | 3.73492119 | 0.00898634 | 0.04348778 | TMEM25 |
| ENSG00000165548 | -2.197214 | 1.31E-06 | 2.24E-05 | TMEM63C |
| ENSG00000165071 | 3.92023734 | 4.72E-07 | 8.97E-06 | TMEM71 |
| ENSG00000006042 | -4.1428581 | 4.19E-35 | 2.54E-32 | TMEM98 |
| ENSG00000184012 | -2.0115383 | 4.66E-26 | 1.21E-23 | TMPRSS2 |
| ENSG00000238164 | -2.544326 | 2.49E-06 | 3.95E-05 | TNFRSF14-AS1 |
| ENSG00000127863 | -6.0856095 | 4.46E-06 | 6.64E-05 | TNFRSF19 |
| ENSG00000239697 | -1.5523598 | 9.15E-11 | 3.56E-09 | TNFSF12 |
| ENSG00000161955 | -1.7569738 | 9.26E-12 | 4.35E-10 | TNFSF13 |
| ENSG00000079308 | -1.5677018 | 5.03E-12 | 2.46E-10 | TNS1 |
| ENSG00000198846 | 1.7368165 | 5.07E-05 | 0.00056165 | TOX |
| ENSG00000124191 | 2.92511689 | 0.00142395 | 0.00976818 | TOX2 |
| ENSG00000227372 | 2.46724294 | 4.09E-33 | 2.07E-30 | TP73-AS1 |
| ENSG00000261594 | 6.4421302 | 4.40E-06 | 6.56E-05 | TPBGL |
| ENSG00000116176 | -1.8341127 | 0.00172619 | 0.01144927 | TPSG1 |
| ENSG00000269113 | 4.118229 | 1.46E-25 | 3.54E-23 | TRABD2B |
| ENSG00000186439 | 3.17549674 | 5.29E-06 | 7.71E-05 | TRDN |
| ENSG00000132274 | -2.3343661 | 0.00419287 | 0.02358569 | TRIM22 |
| ENSG00000137699 | 1.65446225 | 2.52E-06 | 3.98E-05 | TRIM29 |
| ENSG00000227986 | -5.8896744 | 7.69E-05 | 0.00080601 | TRIM60P18 |
| ENSG00000100505 | 2.05979117 | 2.16E-21 | 3.64E-19 | TRIM9 |
| ENSG00000119121 | -2.7945857 | 2.51E-06 | 3.97E-05 | TRPM6 |
| ENSG00000167723 | -2.0478236 | 1.27E-05 | 0.00016682 | TRPV3 |
| ENSG00000165125 | -2.3435311 | 0.00210835 | 0.0135166 | TRPV6 |
| ENSG00000134198 | -2.7887265 | 0.00101564 | 0.0073872 | TSPAN2 |
| ENSG00000127324 | -1.9283737 | 1.15E-36 | 8.06E-34 | TSPAN8 |
| ENSG00000118271 | -1.5603837 | 1.61E-08 | 4.12E-07 | TTR |
| ENSG00000275895 | -3.9265867 | 4.66E-70 | 2.83E-66 | U2AF1L5 |
| ENSG00000242366 | -1.7511821 | 3.86E-28 | 1.21E-25 | UGT1A8 |
| ENSG00000135220 | -1.9158969 | 7.34E-31 | 2.91E-28 | UGT2A3 |
| ENSG00000109181 | -2.3138712 | 1.62E-09 | 4.97E-08 | UGT2B10 |
| ENSG00000213759 | -1.5735047 | 1.62E-05 | 0.00020661 | UGT2B11 |
| ENSG00000196620 | -3.4131219 | 3.04E-44 | 2.91E-41 | UGT2B15 |
| ENSG00000111981 | 6.33723904 | 6.84E-06 | 9.72E-05 | ULBP1 |
| ENSG00000130477 | 1.76908703 | 7.47E-13 | 4.29E-11 | UNC13A |
| ENSG00000100024 | -2.2702647 | 3.15E-09 | 9.26E-08 | UPB1 |
| ENSG00000243566 | -1.5763662 | 0.00215989 | 0.01378397 | UPK3B |
| ENSG00000071246 | 2.20370015 | 0.00039473 | 0.00330664 | VASH1 |
| ENSG00000143494 | 2.02036222 | 0.00381792 | 0.02187502 | VASH2 |
| ENSG00000038427 | -1.7109901 | 8.29E-28 | 2.48E-25 | VCAN |
| ENSG00000136059 | -2.4995841 | 1.29E-32 | 6.17E-30 | VILL |
| ENSG00000109072 | -1.7851578 | 0.00915678 | 0.04414832 | VTN |
| ENSG00000145198 | -1.7794266 | 3.36E-09 | 9.84E-08 | VWA5B2 |
| ENSG00000101443 | 1.8242196 | 0.00806316 | 0.03998659 | WFDC2 |
| ENSG00000127578 | -2.0513782 | 5.08E-06 | 7.45E-05 | WFIKKN1 |
| ENSG00000122574 | 5.59997029 | 1.93E-18 | 2.27E-16 | WIPF3 |
| ENSG00000184937 | 6.19483783 | 7.50E-12 | 3.56E-10 | WT1 |
| ENSG00000255282 | -1.5818096 | 0.00844911 | 0.04151642 | WTAPP1 |
| ENSG00000221947 | -2.0655138 | 0.00973569 | 0.04641139 | XKR9 |
| ENSG00000103489 | -3.7573232 | 0.00806087 | 0.03998611 | XYLT1 |
| ENSG00000102053 | -1.904161 | 3.23E-10 | 1.14E-08 | ZC3H12B |
| ENSG00000126970 | -3.366765 | 2.10E-34 | 1.12E-31 | ZC4H2 |
| ENSG00000166707 | -3.3671007 | 0.00705754 | 0.03586846 | ZCCHC18 |
| ENSG00000179588 | -1.8020771 | 8.30E-11 | 3.27E-09 | ZFPM1 |
| ENSG00000178150 | 2.73393542 | 0.00185233 | 0.01214884 | ZNF114 |
| ENSG00000197935 | 6.96890898 | 1.56E-07 | 3.21E-06 | ZNF311 |
| ENSG00000088876 | 1.6896761 | 1.92E-10 | 7.01E-09 | ZNF343 |
| ENSG00000169981 | -2.2284581 | 0.00426356 | 0.02390951 | ZNF35 |
| ENSG00000144331 | -2.1103962 | 8.77E-06 | 0.0001211 | ZNF385B |
| ENSG00000197013 | -1.6034591 | 0.00023434 | 0.00210643 | ZNF429 |
| ENSG00000197857 | -1.8372568 | 8.40E-09 | 2.29E-07 | ZNF44 |
| ENSG00000197044 | -3.8412919 | 8.10E-07 | 1.45E-05 | ZNF441 |
| ENSG00000168916 | -2.8320723 | 7.07E-13 | 4.13E-11 | ZNF608 |
| ENSG00000196757 | -2.9114165 | 5.23E-20 | 7.81E-18 | ZNF700 |
| ENSG00000234444 | -3.3467896 | 6.29E-13 | 3.70E-11 | ZNF736 |
| ENSG00000239893 | -4.3369457 | 3.50E-06 | 5.34E-05 | ZNF736P9Y |
| ENSG00000105750 | -1.5664997 | 1.36E-05 | 0.00017811 | ZNF85 |
| ENSG00000170044 | -3.6281208 | 0.00249182 | 0.01552702 | ZPLD1 |
