# Supplementary figures and images for: AC093797.1 as a Potential Biomarker to Indicate the Prognosis of Hepatocellular Carcinoma and Inhibits Cell Proliferation, Invasion, and Migration by Reprogramming Cell Metabolism and Extracellular Matrix Dynamics
Source: Front Genet. 2021 Dec 3;12:778742. doi: 10.3389/fgene.2021.778742 (PMC8678093; doi:10.3389/fgene.2021.778742)

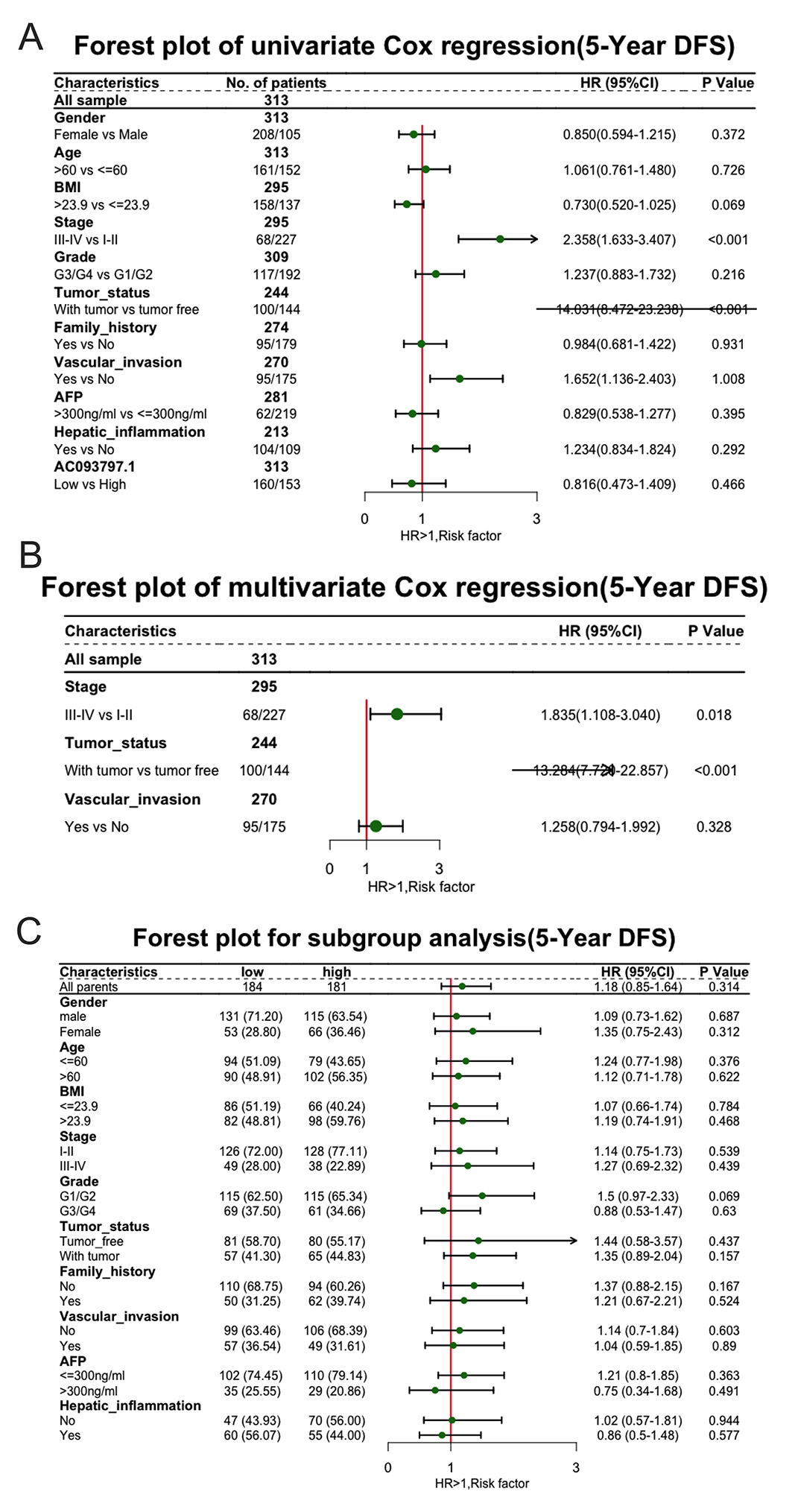

Supplement: Supplementary file 2 [file Image1.TIF]
